# Dual role of N4BP1 in neutrophil–epithelial crosstalk in periodontitis

**DOI:** 10.3389/fimmu.2026.1830039

**Published:** 2026-05-28

**Authors:** Yuqiang Sun, ZiHan Shen, Wenyu Zhen, Fei Xu, Yutong Lu, Yufei Pan, Dawei Mi, Shouzheng Ma, Wei Li, Wansu Sun, Jintao Yu, Hengguo Zhang

**Affiliations:** 1 College & Hospital of Stomatology, Anhui Medical University, Anhui Provincial Key Laboratory of Oral Diseases Research, Hefei, China; 2 Department of Stomatology, Suzhou Hospital of Anhui Medical University, Suzhou, China

**Keywords:** mendelian randomization, N4BP1, neutrophil heterogeneity, periodontitis, single-cell RNAseq

## Abstract

**Background:**

Periodontitis is a chronic inflammatory disease driven by immune dysregulation, in which neutrophils play a central but functionally heterogeneous role. The contributions of specific neutrophil subsets to disease pathogenesis remain poorly defined.

**Methods:**

We integrated single-cell RNA sequencing (scRNA-seq) and Mendelian randomization analysis to identify disease-associated immune cell subsets and gene regulatory mechanisms. Cell-type-specific interactions and pathway alterations were further analyzed to uncover potential pathogenic mediators. Key findings were validated using a murine ligature-induced periodontitis model, supplemented by targeted modulation of N4BP1 in primary human gingival epithelial cells and neutrophils.

**Results:**

We identified a transcriptionally defined neutrophil subset that resembles previously reported low-density neutrophils, which we provisionally designate as LDN-1. This subset is characterized by aberrant interactions with epithelial cells primarily mediated by dysregulation of N4BP1. N4BP1 exhibited a dual, cell−type−specific role: its downregulation in epithelial cells impaired mucosal barrier integrity, leading to increased inflammatory tissue damage. In LDN−1, N4BP1 exhibited a context−dependent regulatory function: it suppresses both pro-inflammatory CXCL1/6-CXCR1/2 signaling and anti-inflammatory ANXA1-FPR1/FPR2 pathways. This duality suggests that N4BP1 may calibrate the balance between pro− and anti−inflammatory signals during periodontitis progression.

**Conclusions:**

Our findings identify N4BP1 as a critical cell-type-specific molecular candidate that coordinates epithelial barrier function and neutrophil activity. Modulating N4BP1 or its downstream pathways may represent a potential precision therapeutic strategy for periodontitis and related inflammatory diseases, pending further interventional validation.

## Graphical Abstract

**Figure:**
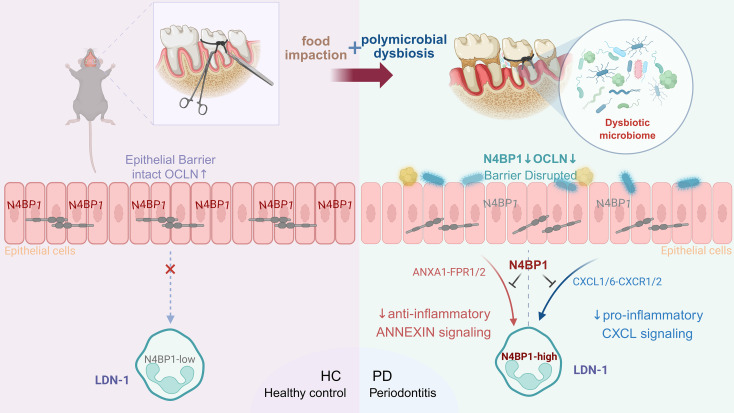
The upper panel depicts the murine periodontitis model induced by silk ligature placement around the maxillary second molar, facilitating food impaction and polymicrobial dysbiosis that elicits inflammatory alveolar bone loss. The lower left panel shows the healthy gingival state: epithelial cells express high levels of N4BP1, which sustains mucosal barrier integrity by preserving tight junction protein occludin (OCLN); the LDN-1 neutrophil subset remains transcriptionally quiescent with negligible epithelial interaction. The lower middle panel illustrates periodontitis (PD): epithelial N4BP1 is downregulated by dysbiotic challenge, resulting in OCLN disruption and compromised barrier function. In contrast, LDN-1 expands and upregulates N4BP1, establishing pathogenic crosstalk with epithelium through two distinct, N4BP1-restricted axes: suppression of the pro-inflammatory chemokine axis CXCL1/6–CXCR1/2 and concomitant inhibition of the anti-inflammatory ANXA1–FPR1/FPR2 resolution pathway. The lower right inset conceptualizes N4BP1 as a cell-type-specific molecular rheostat that simultaneously dampens neutrophil-driven inflammation and endogenous pro-resolving signals, thereby fine-tuning immune–epithelial homeostasis during periodontitis progression. Created with BioRender.com

## Introduction

Periodontitis is a chronic inflammatory disease affecting the periodontium, the supporting structures that anchor teeth to the alveolar bone. It is a highly prevalent condition, clinically presenting with gingival recession, bleeding, and increased tooth mobility, and represents the leading cause of tooth loss and alveolar bone resorption in adults (1, 2).

Typically, periodontitis involves the activation of inflammatory responses triggered by changes in bacterial composition. Among the myriad of oral bacteria, *Porphyromonas gingivalis* is recognized as a keystone pathogen that orchestrates the transition from microbial-host symbiosis to dysbiosis, thereby playing a critical role in disease pathogenesis (3–5). P*. gingivalis* promotes its persistence by adhering to and invading human gingival epithelial cells, while simultaneously subverting innate host responses (6). The initial cellular response to bacterial encounter comprises keratinocytes and stromal fibroblasts, which, though not classical immune cells, play a pivotal role (7, 8). Their crosstalk with immune cells orchestrates gingival inflammation and triggers downstream cascades that lead to connective tissue breakdown. Despite recent advancements offering insights into potential intercellular communications involved, the exact mechanisms underlying this transition are still not fully elucidated.

Neutrophils are polymorphonuclear leukocytes and professional phagocytes of the innate immune system, playing a crucial role as the first line of defense in response to invading pathogens. They are the most abundant white blood cells in humans, constituting 50–70% of circulating leukocytes with an estimated lifespan of up to 5 days (9–11). In recent years, particularly in the contexts of cancer and inflammation, numerous studies have uncovered neutrophil heterogeneity characterized by markedly distinct functional phenotypes. As opposed to the high-density neutrophils (HDNs) which have anti-tumorigenic properties, low-density neutrophils (LDNs) consist of mature and immature neutrophil subsets and have been associated with immunosuppressive functions (12). These evidences have delineated aberrant neutrophil responses in various chronic inflammatory and autoimmune diseases. However, the pathogenic roles and functional heterogeneity of neutrophil subpopulations in periodontitis remain largely unexplored.

Single-cell RNA sequencing (scRNA-seq) enables unbiased transcriptomic profiling of individual cells, revealing cell types, states, and activities through analyses like dimensionality reduction and clustering. Furthermore, Mendelian randomization (MR) is a powerful approach for inferring causal links between exposures and diseases. It offers an alternative to randomized controlled trials (RCTs), which are often limited by cost, ethics, and potential confounding (13). MR helps overcome these issues by using genetic variants, known as single nucleotide polymorphisms (SNPs), which are strongly associated with the traits being studied. This approach helps reduce biases that can occur in traditional RCTs (14). Single-cell technologies and MR provide novel perspectives for molecular studies related to periodontitis.

In this study, we integrated scRNA-seq and MR analyses to dissect the pathogenic role of neutrophil subsets during periodontitis progression. We identified a transcriptionally defined LDN-like subset (LDN-1) that exhibits dynamic, N4BP1-dependent crosstalk with epithelial cells. Strikingly, N4BP1 displayed opposing, cell-type-specific functions: its downregulation in epithelial cells compromised barrier integrity, while its upregulation in LDN-1 was associated with the suppression of both pro-inflammatory (CXCL-CXCR) and pro-resolving (ANXA1-FPR) signaling axes. Our findings establish N4BP1 as a pivotal molecular hub at the immune-epithelial interface and highlight its potential as a therapeutic target for restoring immune homeostasis in periodontitis.

## Results

### Significant cell populations during periodontitis progression and treatment

**Figure 1:**
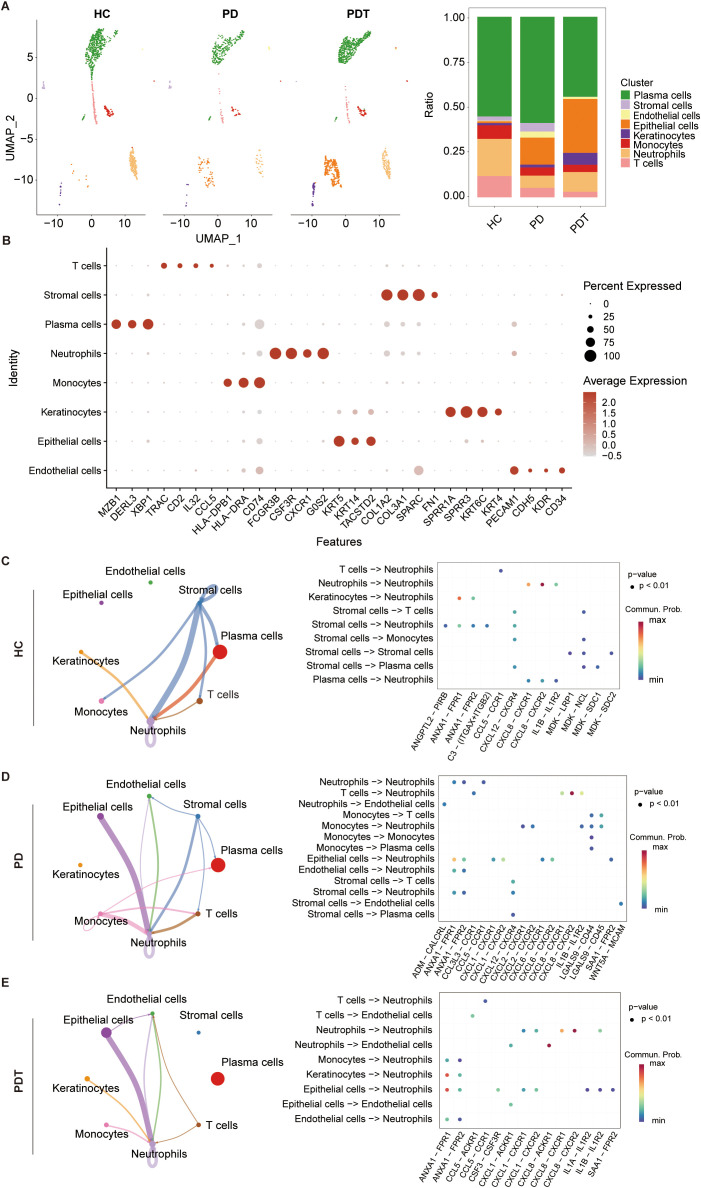
Single-cell atlas reveals altered immune cell proportions and enhanced neutrophil-epithelial interactions in periodontitis. **(A)** UMAP visualization of single-cell transcriptomes from gingival tissues of HC (n=943 cells), PD (n=442 cells), and PDT (n=1,122 cells). Cells are colored by annotated clusters (Left). Bar plots show the proportional distribution of major cell types across groups (Right). **(B)** Dot plots showing the expression patterns of marker genes across the identified cell populations. **(C)** Cell-cell communication inferred from CellPhoneDB analysis of healthy state. **(D)** Cell-cell communication between neutrophils and other cells inferred from CellPhoneDB analysis in periodontitis state. **(E)** Cell-cell communication between neutrophils and other cells inferred from CellPhoneDB analysis in initial periodontitis therapy state. Line thickness indicates the number of signaling pathways between cell types. Dot size and color intensity represent the interaction strength of ligand-receptor pairs. Arrows denote the inferred communication direction (ligand-producing cell → receptor-expressing cell). HC, health control; PD, periodontitis; PDT, periodontal therapy.

After standard data processing and quality filtering, we obtained single-cell transcriptomes from a total of 2507 single cells, including 943 cells from healthy controls (HC, n=4), 442 cells from patients with severe chronic periodontitis (PD, n=5), and 1122 cells from patients with severe chronic periodontitis after initial periodontal therapy within 1 month (PDT, n=3). Unbiased clustering of the cells identified distinct cell populations based on uniform manifold approximation and projection (UMAP) analyses. Through comprehensive marker gene analysis, we annotated eight major cell types: Plasma cells (characterized by MZB1, DERL3, XBP1), T cells (TRAC, CD2, IL32, CCL5), Monocytes (HLA-DPB1, HLA-DRA, CD74), Neutrophils (FCGR3B, CSF3R, CXCR1, G0S2), Epithelial cells (KRT5, KRT14, TACSTD2), Stromal cells (COL1A2, COL3A1, SPARC, FN1), Keratinocytes (SPRR1A, SPRR3, KRT6C, KRT4), and Endothelial cells (PECAM1, CDH5, KDR, CD34) (**Figures 1A, B**). Notably, the proportion of neutrophils was significantly reduced in the PD group compared to the HC group but was partially restored following treatment in the PDT group. In contrast, plasma cell percentages were markedly elevated in the PD group relative to both the HC and PDT groups. Interestingly, epithelial cell proportions increased in the PD group compared to the HC group and rose even further after treatment. To exclude potential bias arising from unequal cell numbers across groups (HC: 943 cells; PD: 442 cells; PDT: 1,122 cells), we performed downsampling sensitivity analyses. By randomly subsampling HC and PDT to match the PD group size (n = 442) over 1,000 repetitions, we confirmed that the proportional distributions of epithelial and neutrophil populations remained consistent with the original data (**Supplementary Figures 1A–D**). Biologically, the increased epithelial proportion in PD may reflect inflammation-induced basal cell proliferation and barrier regenerative responses, whereas the concomitant decrease in neutrophil proportion likely reflects transmigration into periodontal tissues and NETosis. Partial recovery of the neutrophil percentage in the PDT group indicates restoration of the circulating neutrophil pool. Furthermore, detection probability analysis confirmed that with the total neutrophil count present in PD, the probability of detecting the LDN-1 subset was 100% (**Supplementary Figure 2**), supporting the reliable identification of this subset.

Next, we respectively utilized CellPhoneDB to identify the ligand-receptor pairs and molecular interactions within the primary cell types (**Figures 1C–E**). The circle plot detected broadcast ligands and demonstrated extensive communication for cognate receptors. It is noteworthy that while there was no interaction between epithelial cells and neutrophils in the HC group, the interaction between these two cell types was most pronounced in the PD group. Interestingly, the interactive pathway in the PDT group even surpassed that of the PD group. In particular, stromal cells exhibited the highest level of interaction with neutrophils in the HC group. Notably, in the PD group, epithelial cells predominantly interacted with neutrophils through the ANAX1-FPR1/FPR2, CXCL1-CXCR1/CXCR2, and CXCL6-CXCR1/CXCR2 signaling pathways (**Figure 1D**). In summary, the percentage of neutrophils was significantly decreased in the periodontitis disease groups and demonstrated a significant interaction with epithelial cells, suggesting that neutrophils may function in the progression of periodontitis through their interaction with epithelial cells.

### In-depth analysis of the functions of neutrophil cluster subsets

**Figure 2:**
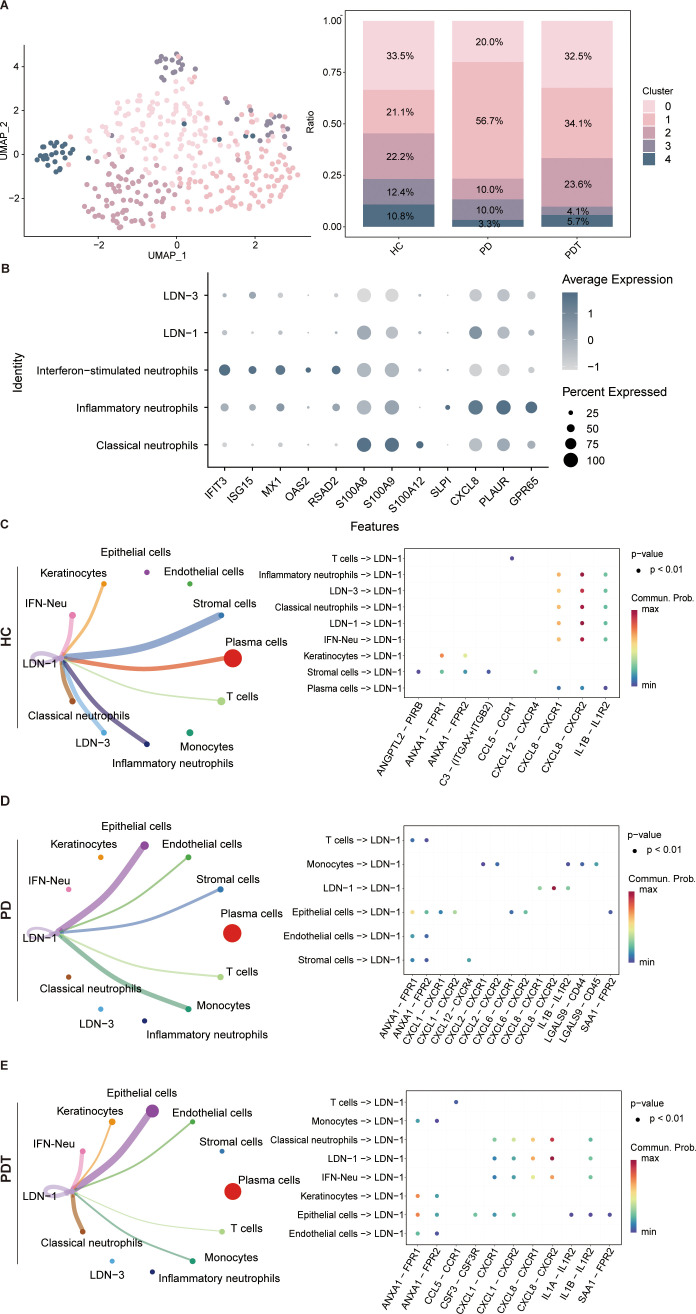
Heterogeneity of LDN-1 and its selective interaction with epithelial cells in periodontitis. **(A)** UMAP visualization of neutrophil subclustering reveals five distinct neutrophil subsets (LDN-0 to LDN-4) (Left). Bar plots show proportional distribution of neutrophil subsets across groups (Right). **(B)** Dot plots illustrating the expression of marker genes across three neutrophil subsets: Interferon-stimulated neutrophils, Classical neutrophils, and Inflammatory neutrophils. **(C)** Cell-cell communication across cell types inferred from CellPhoneDB analysis in healthy state. **(D)** Cell-cell communication between LDN-1 and epithelial cells inferred from CellPhoneDB analysis in periodontitis state. **(E)** Cell-cell communication between LDN-1 and epithelial cells inferred from CellPhoneDB analysis in initial periodontitis therapy state. Line thickness indicates the number of signaling pathways between cell types. Dot size and color intensity represent the interaction strength of ligand-receptor pairs. Arrows denote the inferred communication direction (ligand-producing cell → receptor-expressing cell). IFN-Neu, Interferon-stimulated neutrophils; HC, health control; PD, periodontitis; PDT, periodontal therapy.

To identify which neutrophil subset drives this enhanced interaction, we conducted further research and analysis on the subclusters of neutrophils. We identified five neutrophil clusters, which were then annotated based on the characteristic expression of cluster-specific marker genes using the online tools CellMarker 2.0 and SingleR (**Figures 2A, B**). Clusters 0, 2, and 4 were annotated as Interferon-stimulated neutrophils, Classical neutrophils, and Inflammatory neutrophils, respectively. As no established neutrophil subsets corresponding to clusters 1 and 3 could be identified using canonical markers, we annotated these clusters via the SingleR reference−based tool, which suggested transcriptional similarity to previously reported low−density neutrophils (LDNs). Because physical density gradient isolation was not performed in this study, we cautiously refer to these as transcriptionally defined LDN−like subsets, and for clarity we redesignate them as LDN−1 and LDN−3, respectively, throughout the subsequent analyses. Compared to the HC group, the proportion of LDN-1 was significantly increased in the PD group but partially recovered after initial periodontal treatment. Intriguingly, the upregulation proportion of this subset in the PD group exceeded that of the HC group by over 30%. Other subsets exhibited a downregulation trend in the PD group.

To investigate the mechanistic underpinnings of cellular crosstalk within the neutrophil-epithelial axis, we first integrated five subsets of neutrophil subpopulations with other cellular populations into a single dataset. We then utilized CellPhoneDB to identify ligand-receptor pairs among these cell types. As shown in **Figures 2C–E**, only the LDN-1 exhibited pathway interactions with epithelial cells in the PD group. Based on these interaction results, along with the observed neutrophil-epithelial cell interactions shown in **Figure 1D**, it is suggested that LDN-1 may represent a key cellular subpopulation mediating neutrophil-epithelial crosstalk in periodontitis. Following initial periodontal treatment, Interferon-stimulated neutrophils and Classical neutrophils also demonstrated interactions with epithelial cells. Additionally, Inflammatory neutrophils in three groups lacked interactions with epithelial cells. In summary, the aforementioned results indicate that the LDN-1 interacts with epithelial cells during the progression of periodontitis.

### Identification of potential genes in periodontitis

**Figure 3:**
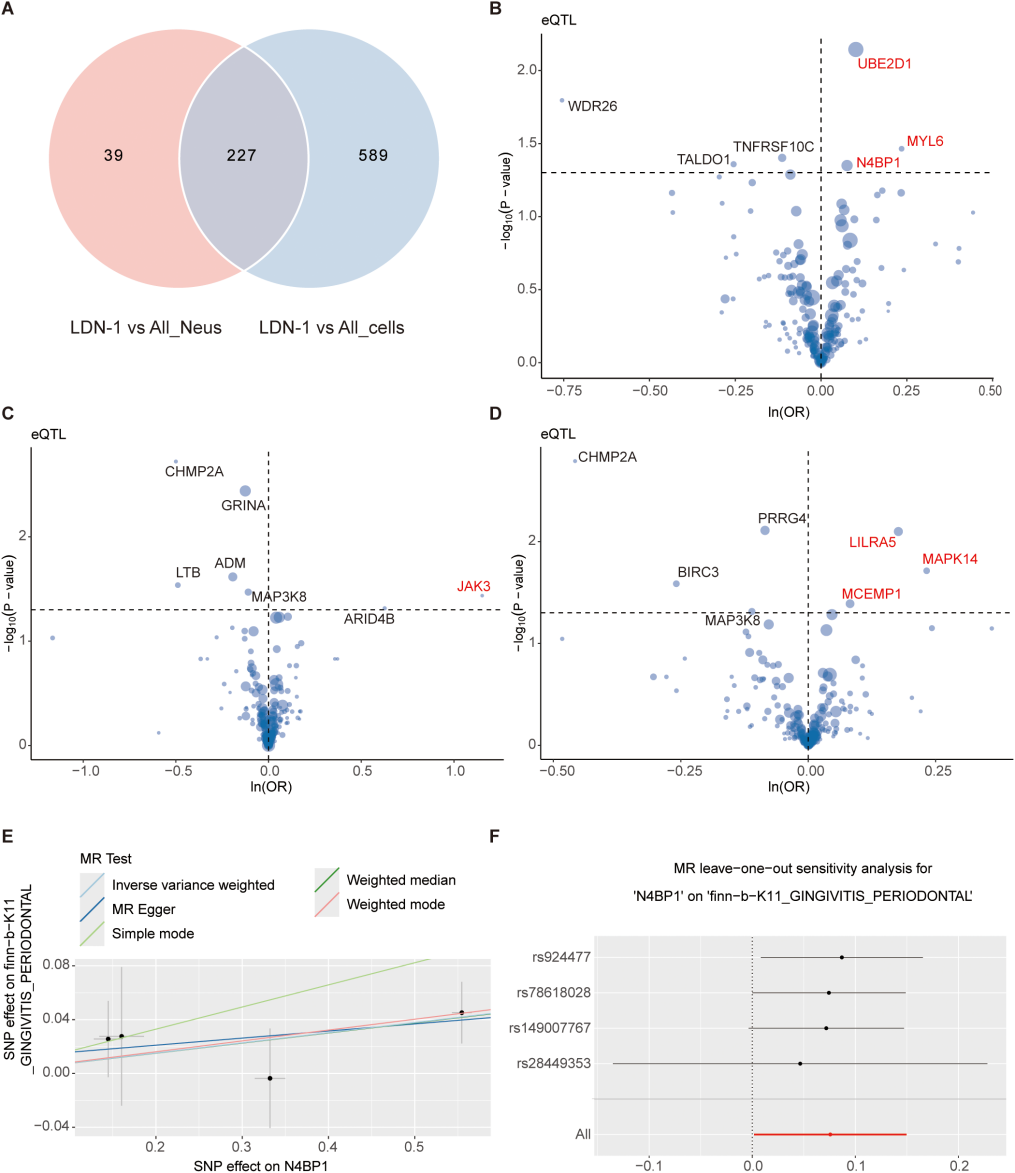
Potential pathogenic regulators of periodontitis identified by integrated differential expression and mendelian randomization analyses. **(A)** Venn diagram showing overlap of differentially expressed genes between LDN-1 versus other neutrophil subpopulations (266 genes) and LDN-1 versus all other cells (816 genes), yielding 227 candidate genes. **(B)** Volcano maps for MR analysis of candidate genes on the finn-b-K11_GINGIVITIS_PERIODONTAL dataset. **(C)** Volcano maps for MR analysis of candidate genes on the ebi-a-GCST90018677 dataset. **(D)** Volcano maps for MR analysis of candidate genes on the ebi-a-GCST90018897 dataset. Genes are plotted by causal effect size (ln[OR]) versus statistical significance (-log_10_[P-value]). Highlighted genes (red) show significant causal relationships with periodontitis (IVW p < 0.05). **(E)** Scatter plot of SNP effects on N4BP1 expression versus periodontitis. **(F)** Leave-one-out sensitivity analysis for N4BP1. Each point represents the IVW estimate when excluding one SNP, confirming no single variant drives the association. MR, mendelian randomization; OR, odds ratio; IVW, inverse variance weighted; SNP, single nucleotide polymorphism.

The phenotypic alterations observed in the LDN-1 underscore its pivotal role in periodontitis. We identified 266 differentially expressed genes in the LDN-1 population compared to other neutrophil subtypes. Following analysis of the LDN-1 population versus other cellular populations, including Plasma cells, T cells, and Epithelial cells, we obtained 816 differentially expressed genes, which were derived through the FindMarkers function. By intersecting these differential gene sets, we ultimately identified 227 genes differentially expressed in LDN-1 (**Figure 3A**).

Based on our MR screening criteria, we identified seven genes that exhibit significant causal relationships with periodontitis (**Figures 3B–D**; **Table 1**). According to the results from the IVW method, the six potential risk genes are UBE2D1 (*p* = 0.0072, OR = 1.1067, 95%CI = 1.0278 – 1.1916), MYL6 (*p* = 0.0343, OR = 1.2646, 95%CI = 1.0175 – 1.5717), N4BP1 (*p* = 0.0447, OR = 1.0787, 95%CI = 1.0018 – 1.1614), MAPK14 (*p* = 0.0193, OR = 1.2615, 95%CI = 1.0383 – 1.5326), MCEMP1 (*p* = 0.0407, OR = 1.0855, 95%CI = 1.0035 – 1.1743), LILRA5 (*p* = 0.0080, OR = 1.1934, 95%CI = 1.0473 – 1.3599), JAK3 (*p* = 0.0366, OR = 3.1646, 95%CI = 1.0745 - 9.3207). To provide a comprehensive overview of the MR analysis, we have included the results of different methods in **Table 1**. Although JAK3 exhibited statistical significance in the MR analysis (p < 0.05), it was deemed unsuitable for further consideration due to methodological limitations, including the presence of only a single instrumental variable and the absence of sensitivity analyses, which compromised the robustness and applicability of the findings. Therefore, it was not considered as our primary research focus. It is important to note that these MR findings are intended to serve as hypothesis-generating evidence rather than definitive proof of causality. The modest effect size observed for N4BP1 (IVW OR = 1.0787, 95% CI: 1.0018–1.1614, p = 0.0447) is consistent with complex inflammatory disease genetics and justified its prioritization for downstream multi-modal validation. The concordance between scatterplot visualization and leave-one-out pleiotropy assessments strengthened confidence in the causal inferences derived from MR analyses (**Figures 3E, F**; **Supplementary Figure 3**). Furthermore, the Cochran’s Q test and MR-Egger did not reveal any evidence of heterogeneity or horizontal pleiotropy (**Tables 2**, **3**). Nevertheless, subsequent integrative single-cell co-expression analysis and directed functional perturbation experiments further supported this causal inference.

**Table 1:** MR analysis results of potentially pathogenic regulatory factors for periodontitis.

Outcome	Exposure	MR method	No. of SNP	SE	*P*-value	OR (95% CI)
finn-b-K11_GINGIVITIS_PERIODONTAL	N4BP1	MR Egger	4	0.0829	0.5893	1.0542 (0.8961 - 1.2402)
finn-b-K11_GINGIVITIS_PERIODONTAL	N4BP1	Weighted median	4	0.0378	0.0464	1.0782 (1.0012 - 1.1610)
finn-b-K11_GINGIVITIS_PERIODONTAL	N4BP1	Inverse variance weighted	4	0.0377	0.0447	1.0787 (1.0018 - 1.1614)
finn-b-K11_GINGIVITIS_PERIODONTAL	N4BP1	Simple mode	4	0.0649	0.0849	1.1790 (1.0382 - 1.3389)
finn-b-K11_GINGIVITIS_PERIODONTAL	N4BP1	Weighted mode	4	0.0390	0.1306	1.0840 (1.0042 - 1.1702)
finn-b-K11_GINGIVITIS_PERIODONTAL	UBE2D1	MR Egger	7	0.0632	0.3215	1.0720 (0.9471 - 1.2134)
finn-b-K11_GINGIVITIS_PERIODONTAL	UBE2D1	Weighted median	7	0.0404	0.0135	1.1050 (1.0209 - 1.1961)
finn-b-K11_GINGIVITIS_PERIODONTAL	UBE2D1	Inverse variance weighted	7	0.0377	0.0072	1.1067 (1.0278 - 1.1916)
finn-b-K11_GINGIVITIS_PERIODONTAL	UBE2D1	Simple mode	7	0.0602	0.1252	1.1131 (0.9893 - 1.2524)
finn-b-K11_GINGIVITIS_PERIODONTAL	UBE2D1	Weighted mode	7	0.044	0.0658	1.1050 (1.0128 - 1.2055)
finn-b-K11_GINGIVITIS_PERIODONTAL	MYL6	MR Egger	3	0.2004	0.3252	1.4298 (0.9654 - 2.1177)
finn-b-K11_GINGIVITIS_PERIODONTAL	MYL6	Weighted median	3	0.1200	0.0423	1.2759 (1.0085 - 1.6143)
finn-b-K11_GINGIVITIS_PERIODONTAL	MYL6	Inverse variance weighted	3	0.1109	0.0343	1.2646 (1.0175 - 1.5717)
finn-b-K11_GINGIVITIS_PERIODONTAL	MYL6	Simple mode	3	0.1839	0.5242	1.1510 (0.8027 - 1.6505)
finn-b-K11_GINGIVITIS_PERIODONTAL	MYL6	Weighted mode	3	0.1290	0.1558	1.3329 (1.0351 - 1.7164)
ebi-a-GCST90018897	MAPK14	MR Egger	3	0.2037	0.3506	1.3933 (0.9346 - 2.0772)
ebi-a-GCST90018897	MAPK14	Weighted median	3	0.1107	0.0444	1.2491 (1.0055 - 1.5516)
ebi-a-GCST90018897	MAPK14	Inverse variance weighted	3	0.0993	0.0193	1.2615 (1.0383 - 1.5326)
ebi-a-GCST90018897	MAPK14	Simple mode	3	0.1522	0.4959	1.1339 (0.8414 - 1.5280)
ebi-a-GCST90018897	MAPK14	Weighted mode	3	0.1362	0.4139	1.1495 (0.8802 - 1.5014)
ebi-a-GCST90018897	MCEMP1	MR Egger	8	0.0615	0.7179	1.0235 (0.9074 - 1.1546)
ebi-a-GCST90018897	MCEMP1	Weighted median	8	0.0440	0.1453	1.0662 (0.9781 - 1.1624)
ebi-a-GCST90018897	MCEMP1	Inverse variance weighted	8	0.0401	0.0407	1.0855 (1.0035 - 1.1743)
ebi-a-GCST90018897	MCEMP1	Simple mode	8	0.1208	0.4405	1.1038 (0.8711 - 1.3987)
ebi-a-GCST90018897	MCEMP1	Weighted mode	8	0.0466	0.2380	1.0620 (0.9693 - 1.1636)
ebi-a-GCST90018897	LILRA5	MR Egger	4	0.0904	0.1780	1.2027 (1.0074 - 1.4360)
ebi-a-GCST90018897	LILRA5	Weighted median	4	0.0700	0.0061	1.2117 (1.0564 - 1.3899)
ebi-a-GCST90018897	LILRA5	Inverse variance weighted	4	0.0666	0.0080	1.1934 (1.0473 - 1.3599)
ebi-a-GCST90018897	LILRA5	Simple mode	4	0.1051	0.1676	1.2098 (0.9846 - 1.4865)
ebi-a-GCST90018897	LILRA5	Weighted mode	4	0.0749	0.0815	1.2136 (1.0478 - 1.4055)
ebi-a-GCST90018677	JAK3	Wald ratio	1	0.5511	0.0366	3.1646 (1.0745 - 9.3207)

SNP, single nucleotide polymorphism; SE, standard error; OR, odds ratio; CI, confidence interval. *P*<0.05 was considered statistically significant.

**Table 2:** Results of the Cochran’s Q test for potentially pathogenic regulatory factors in periodontitis.

Outcome	Exposure	Method	Q_pval
finn-b-K11_GINGIVITIS_PERIODONTAL	N4BP1	MR Egger	0.642435
finn-b-K11_GINGIVITIS_PERIODONTAL	N4BP1	Inverse variance weighted	0.805733
finn-b-K11_GINGIVITIS_PERIODONTAL	UBE2D1	MR Egger	0.698919
finn-b-K11_GINGIVITIS_PERIODONTAL	UBE2D1	Inverse variance weighted	0.757107
finn-b-K11_GINGIVITIS_PERIODONTAL	MYL6	MR Egger	0.674822
finn-b-K11_GINGIVITIS_PERIODONTAL	MYL6	Inverse variance weighted	0.698507
ebi-a-GCST90018897	MAPK14	MR Egger	0.381202
ebi-a-GCST90018897	MAPK14	Inverse variance weighted	0.583012
ebi-a-GCST90018897	MCEMP1	MR Egger	0.480946
ebi-a-GCST90018897	MCEMP1	Inverse variance weighted	0.420306
ebi-a-GCST90018897	LILRA5	MR Egger	0.698628
ebi-a-GCST90018897	LILRA5	Inverse variance weighted	0.865315

Q_pval < 0.05 indicates significant heterogeneity.

**Table 3:** Horizontal pleiotropic analysis results of potentially pathogenic regulatory factors in periodontitis.

Outcome	Exposure	Egger_intercept	SE	*P*-value
finn-b-K11_GINGIVITIS_PERIODONTAL	UBE2D1	0.013298658	0.021188	0.557771
finn-b-K11_GINGIVITIS_PERIODONTAL	MYL6	-0.020716082	0.028149	0.596102
finn-b-K11_GINGIVITIS_PERIODONTAL	N4BP1	0.010454173	0.033653	0.785456
ebi-a-GCST90018897	MAPK14	-0.017779509	0.031816	0.675588
ebi-a-GCST90018897	MCEMP1	0.016139455	0.012845	0.255663
ebi-a-GCST90018897	LILRA5	-0.002497663	0.019654	0.910498

SE, standard error. The intercept term from MR-Egger regression. *P*-value < 0.05 indicates significant horizontal pleiotropy.

### The involvement of potential genes in expression

**Figure 4:**
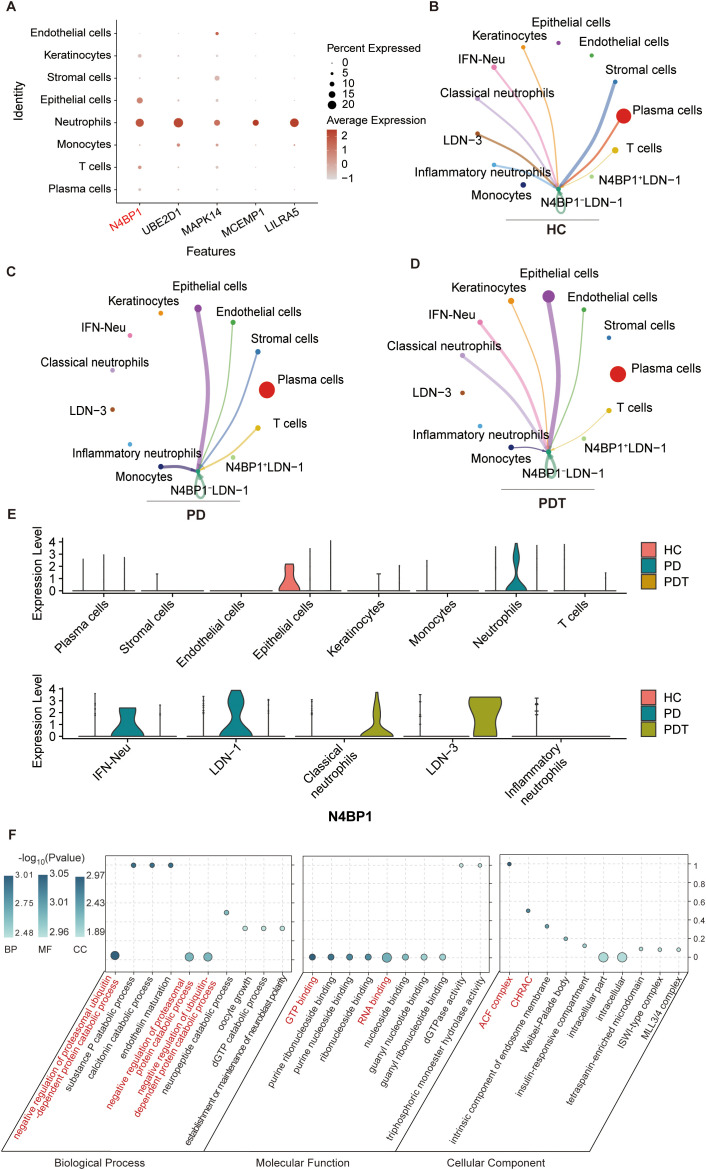
Cell-type-specific expression and functional characterization of N4BP1 in periodontitis. **(A)** Dot plot showing expression of candidate genes (N4BP1, UBE2D1, MAPK14, MCEMP1, LILRA5) across major cell types. **(B)** Cell-cell communication networks between N4BP1^+^/^-^ LDN-1 and epithelial cells in healthy state. **(C)** Cell-cell communication networks between N4BP1^+^/^-^ LDN-1 and epithelial cells in periodontitis state. **(D)** Cell-cell communication networks between N4BP1^+^/^-^ LDN-1 and epithelial cells in initial periodontitis therapy state. Line thickness indicates the number of signaling pathways between cell types. **(E)** Violin plots of N4BP1 expression across cell clusters. Note that the limited number of LDN−1 cells in the PD group (n=17) precluded robust statistical comparison; the observed trend of increased expression was consistent with the functional role of N4BP1 in neutrophil regulation (see text). **(F)** GO enrichment analysis of N4BP1-associated functions. IFN-Neu, Interferon-stimulated neutrophils; GO, gene ontology.

After identifying the potential genes, we prioritized investigating their expression in all cells. As illustrated in the **Figure 4A**, N4BP1 is predominantly expressed in neutrophils and epithelial cells. In the HC group, N4BP1 expression was mainly localized within epithelial cells. In the PD group, N4BP1 was also detected in neutrophils, particularly within the LDN-1 (**Figure 4E**). The remaining genes, including UBE2D1, MAPK14, MCEMP1, and LILRA5, are primarily expressed in neutrophils (**Supplementary Figures 4A–D**). In which, there are no significant differences in the expression of MAPK14, MCEMP1, and LILRA5 between the HC and PD groups (**Supplementary Figures 4B–D**). In contrast, UBE2D1 expression is downregulated in the PD group (**Supplementary Figure 4A**). Meanwhile, MYL6 exhibits high expression across all cell types examined in this study, with no significant differences observed between the HC and PD groups (**Supplementary Figure 4E**). Our findings suggest that N4BP1 is highly likely to promote the pathogenic role of the neutrophil-epithelial cell axis in periodontitis.

### Molecular mechanisms of N4BP1

To identified the role of the N4BP1, we investigated its heterogeneous expression patterns and signaling pathways in positive and negative groups. We examined the dynamic interactions between N4BP1(+/-) positive and negative groups of LDN-1 and other cells (**Figures 4B–D**). Comparative analysis revealed that N4BP1 significantly inhibits the communication between epithelial cells and LDN-1. Furthermore, we investigated differentially expressed genes in the positive and negative groups and performed enrichment analysis. We identified N4BP1 is primarily involved in the Biological Processes, such as negative regulation of proteasomal ubiquitin-dependent protein catabolic process, substance P catabolic process, calcitonin catabolic process, endothelin maturation, negative regulation of proteasomal protein catabolic process and negative regulation of ubiquitin-dependent protein catabolic process (**Figure 4F**). The GO cellular component (GOCC) enrichment analysis revealed significant associations of N4BP1 with chromatin remodeling pathways, particularly through its interaction with the ACF complex (15) and CHRAC (16). This nuclear localization pattern strongly suggests that N4BP1 exerts its biological functions within the nuclear compartment. Furthermore, molecular function (GOMF) characterization demonstrated that N4BP1 primarily engages in nucleic acid-binding activities, with notable enrichment in GTP binding, purine ribonucleoside triphosphate binding, and RNA binding. These coordinated findings substantiate the dual functional capacity of N4BP1 in both chromatin structural modulation and nucleic acid interaction processes. KEGG pathway enrichment analysis revealed significant associations of N4BP1 with ferroptosis (ko04216), apoptosis (ko04214), mammalian mitophagy (ko04137), and lysine degradation pathways (ko00310) (**Supplementary Figure 5**). Our findings indicate that N4BP1 disrupts the neutrophil-epithelial cell interaction and predominantly exerts a negative regulatory function.

### Validation of N4BP1 expression heterogeneity in epithelial cells and neutrophils during periodontitis progression

**Figure 5:**
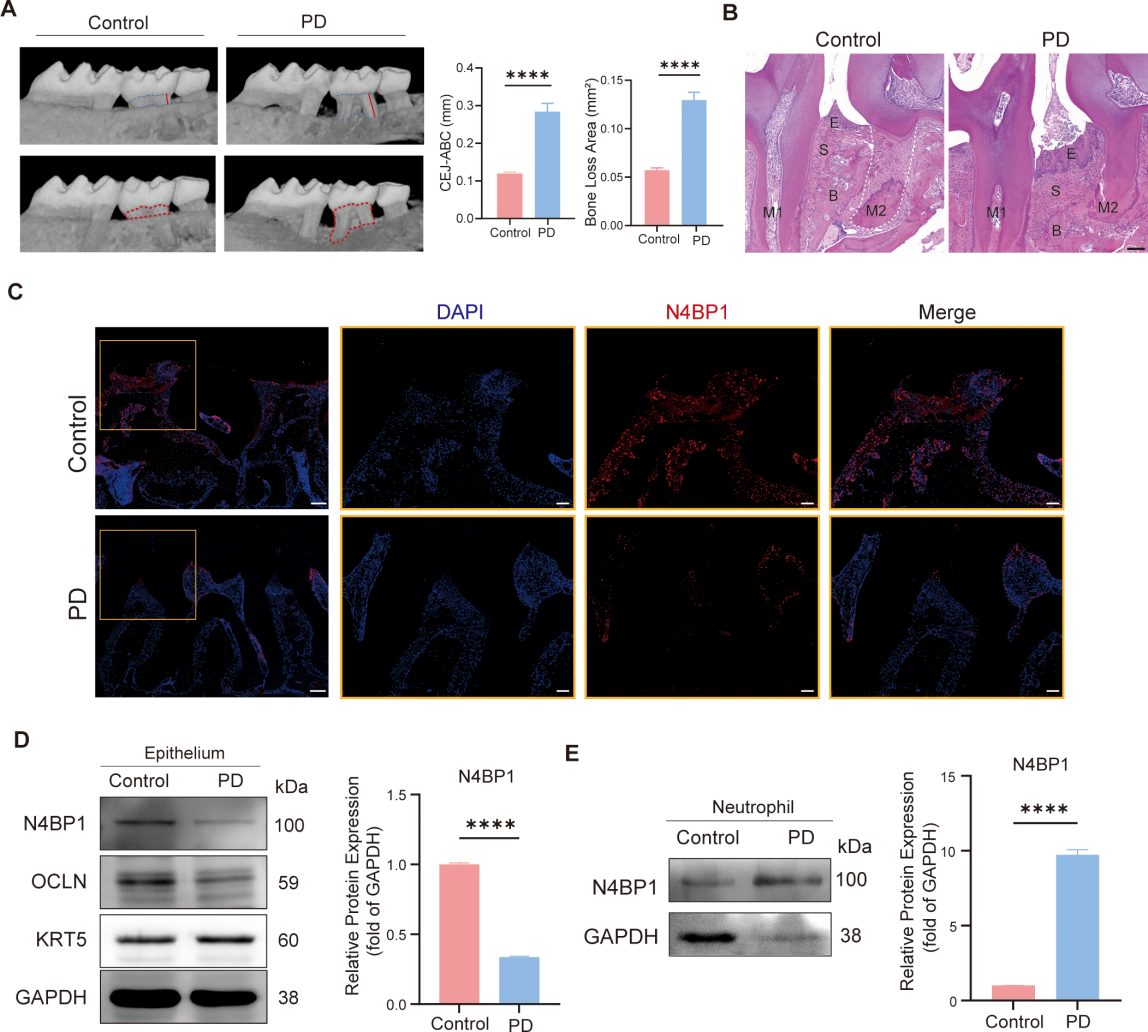
*In vitro* validation of N4BP1 expression heterogeneity in epithelial cells and neutrophils during periodontitis progression. **(A)** Representative micro-CT images and 3D reconstructions of the maxillae from control and periodontitis (PD) mice. The red lines indicate the distance between the cemento-enamel junction (CEJ) and the alveolar bone crest (ABC). The red dotted outline highlights the region of bone resorption, with the upper and lower blue lines corresponding to the CEJ and ABC, respectively. Quantitative analysis of CEJ-ABC distance and bone loss area is shown on the right. Data are presented as mean ± SEM. ****p < 0.0001. **(B)** Representative H&E staining of gingival tissues from control and periodontitis mice. E, epithelial layer; S, stromal layer; B, interdental alveolar bone. Scale bars: 100 μm. **(C)** Immunofluorescence staining of N4BP1 in gingival tissues from control and periodontitis mice. Scale bar: 100 μm. **(D)** Western blot analysis of N4BP1 expression in epithelial cells. OCLN, occludin. Data are presented as mean ± SEM. ****p < 0.0001. **(E)** Western blot analysis of N4BP1 expression in neutrophils. Data are presented as mean ± SEM. ****p < 0.0001.

To corroborate the regulatory role of N4BP1, we conducted systematic *in vivo* experiments using a murine periodontitis model. Ligature-induced periodontitis was successfully established in mice by placing silk sutures around the maxillary right second molars for 14 days. Micro-CT analysis and 3D reconstructed images revealed significant alveolar bone resorption in periodontitis-affected mice (**Figure 5A**, left panel). Furthermore, targeted measurements of the right maxillary second molar demonstrated increased cementoenamel junction-to-alveolar bone crest (CEJ-ABC) distance and buccal bone loss area compared to controls (**Figure 5A**, right panel). Histological analysis revealed that resorption of alveolar bone crest and gingival papilla was more severe in the periodontitis group compared to the control group (**Figure 5B**). Immunofluorescence staining of the inflamed region between the first and second molars similarly confirmed that N4BP1 expression was downregulated in the periodontitis group (**Figure 5C**). Western blot analysis of gingival epithelial tissues revealed a significant downregulation of N4BP1 in the periodontitis group compared to controls (**Figure 5D**). Concurrently, occludin (OCLN) (17) were markedly reduced, while KRT5 was upregulated in diseased tissues, indicating compromised epithelial barrier integrity during periodontitis. These findings align with the scRNA-seq data, suggesting that N4BP1 depletion in epithelial cells exacerbates tissue vulnerability to inflammatory damage.

In contrast to epithelial cells, neutrophils exhibited elevated N4BP1 expression in the disease group (**Figure 5E**). This cell-type-specific expression pattern underscores the dual regulatory role of N4BP1: suppressing epithelial inflammation while enhancing neutrophil-mediated immune modulation.

### Cell-cell communication profiling identifies N4BP1-associated signaling axes in neutrophil-epithelial crosstalk

**Figure 6:**
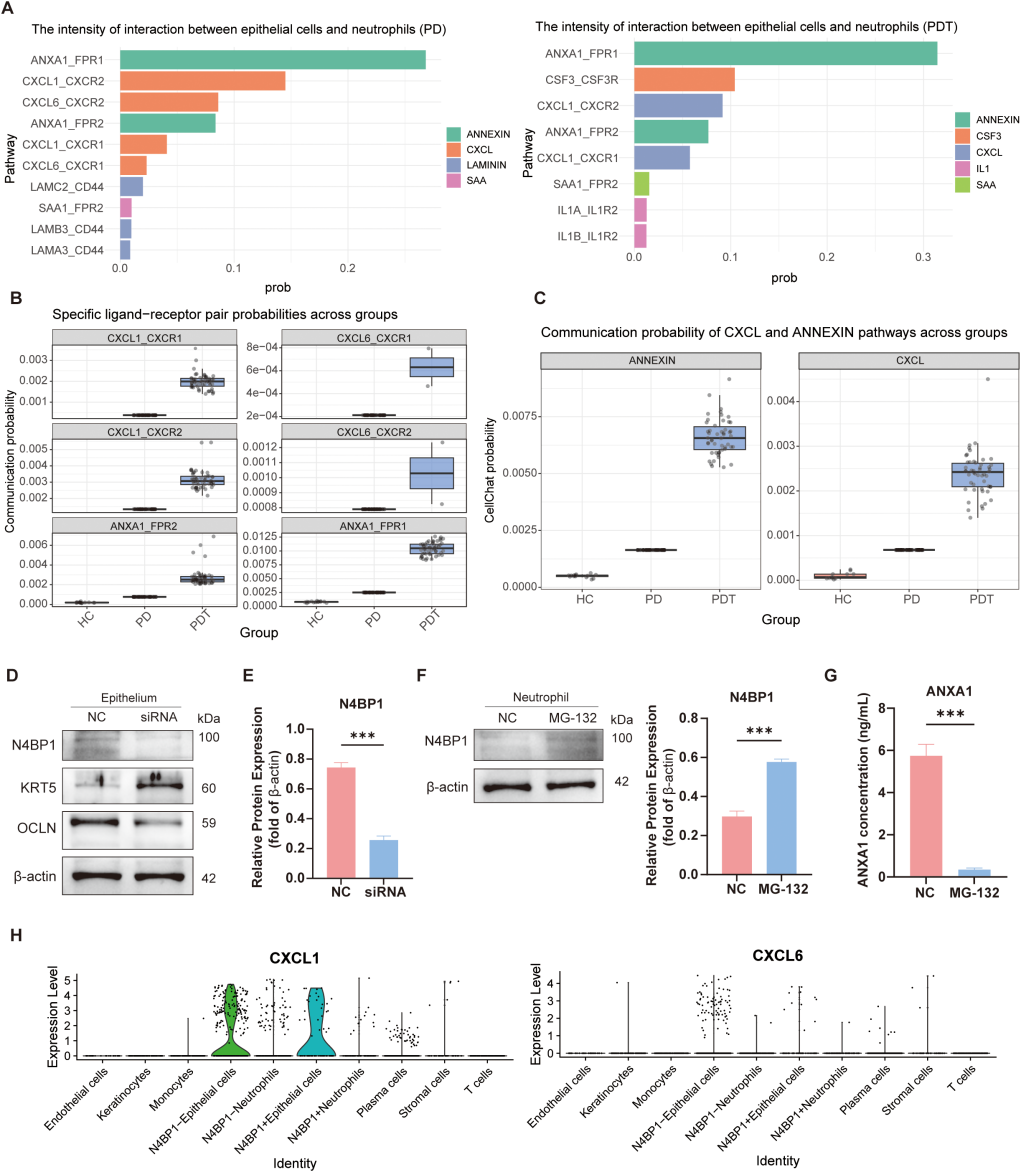
Functional validation of N4BP1 in epithelial barrier maintenance and neutrophil signaling. **(A)** Interaction intensity histogram between epithelial cells and neutrophils in PD and PDT groups, analyzed by CellPhoneDB. Signaling pathways are color-coded by family: ANNEXIN (green), CXCL (red), LAMININ (blue), and SAA (purple). Left: PD group; Right: PDT group. **(B)** Box plots showing specific ligand-receptor pair probabilities across HC, PD and PDT groups. Communication probabilities for CXCL1/6-CXCR1/2 and ANXA1-FPR1/2 axes are significantly elevated in PD compared to HC, with partial attenuation in PDT. Each dot represents an individual sample; box boundaries indicate 25th and 75th percentiles; horizontal lines denote medians. **(C)** Sensitivity analysis of communication probability for ANNEXIN (left) and CXCL (right) pathways across HC, PD, and PDT groups. Box plots illustrate aggregated pathway-level communication probabilities derived from CellChat analysis. **(D, E)** N4BP1 knockdown in primary human gingival epithelial cells (PGECs). **(D)** Representative Western blots showing N4BP1, tight junction protein occludin (OCLN), keratin 5 (KRT5), and β-actin loading control following siRNA-mediated N4BP1 silencing. **(E)** Quantification of N4BP1 protein levels normalized to β-actin. Data represent mean ± SEM from three independent experiments; ***P < 0.001 by unpaired two-tailed Student’s t-test. **(F, G)** Pharmacological upregulation of N4BP1 in primary human neutrophils. Cells were treated with the proteasome inhibitor MG-132 (20 μM, 3 h) to prevent N4BP1 degradation. **(F)** Western blot confirmation of elevated N4BP1 protein levels following MG-132 treatment. **(G)** Secreted ANXA1 concentration in culture supernatants measured by ELISA. N4BP1 stabilization significantly attenuates ANXA1 secretion. Data represent mean ± SEM from three independent donors; ***P < 0.001 by unpaired two-tailed Student’s t-test. NC, negative control (scrambled siRNA for **(E, F)**; DMSO vehicle for **(F, G)**). **(H)** Violin plots depicting expression levels of CXCL1 **(left)** and CXCL6 (right) across cell populations. N4BP1^+^ epithelial cells and neutrophils exhibit attenuated chemokine expression compared to N4BP1^-^ counterparts, suggesting N4BP1-mediated suppression of pro-inflammatory chemokine production.

To delineate the molecular underpinnings of neutrophil–epithelial interactions in periodontitis, we first interrogated ligand–receptor communication probabilities using CellPhoneDB and CellChat analyses. Notably, the PDT group exhibited even stronger epithelial–neutrophil interaction probabilities than the PD group. CellChat pathway analysis revealed that this quantitative enhancement was accompanied by a qualitative shift: PDT was characterized by elevated ANNEXIN and CSF3 signaling, whereas PD was dominated by CXCL1/6–CXCR1/2 and ANXA1–FPR1/2 axes (**Figure 6A**). Quantitative analysis of specific ligand–receptor pair probabilities across HC, PD, and PDT groups revealed that CXCL1/6–CXCR1/2 and ANXA1–FPR1/2 communication probabilities were significantly elevated in PD compared to HC, with partial attenuation following periodontal therapy (**Figure 6B**; **Supplementary Figure 6**). Sensitivity analysis at the pathway level confirmed that both ANNEXIN and CXCL signaling exhibited disease-associated activation patterns, peaking in PD and showing intermediate levels in PDT (**Figure 6C**). These observations establish that neutrophil–epithelial crosstalk in periodontitis is orchestrated through a defined set of N4BP1-responsive signaling axes, providing a mechanistic framework for subsequent functional validation.

### N4BP1 directly controls epithelial barrier integrity in primary gingival epithelial cells

To determine whether the reduction of N4BP1 observed in periodontitis epithelium directly contributes to barrier dysfunction, we knocked down N4BP1 in primary human gingival epithelial cells (PGECs) using specific siRNA. Western blot analysis confirmed N4BP1 silencing (**Figures 6D, E**). Notably, N4BP1 knockdown led to a significant decrease in occludin (OCLN) protein levels and a marked increase in keratin 5 (KRT5) expression (**Figure 6D**), phenocopying the alterations previously found in periodontitis tissues. These results demonstrate that N4BP1 loss is sufficient to disrupt epithelial barrier integrity, supporting its role in maintaining the gingival epithelial barrier.

### N4BP1 differentially regulates ANXA1 and chemokine pathways in neutrophils

To functionally dissect the role of N4BP1 in neutrophil-epithelial crosstalk, we pharmacologically stabilized N4BP1 protein in primary human neutrophils using MG-132, a proteasome inhibitor that prevents N4BP1 degradation (18). Western blot analysis confirmed efficient elevation of endogenous N4BP1 levels following MG-132 treatment (**Figure 6F**). Notably, N4BP1 stabilization significantly reduced the secretion of Annexin A1 (ANXA1), an anti-inflammatory mediator that acts via FPR1/FPR2, as measured by ELISA (**Figure 6G**). Consistent with this functional observation, single−cell transcriptomic analysis revealed that N4BP1^+^ epithelial cells and neutrophils exhibited lower expression of the pro−inflammatory chemokines CXCL1 and CXCL6 compared with their N4BP1^-^ counterparts (**Figure 6H**). Although direct experimental evidence for N4BP1−mediated CXCL6 regulation is currently lacking, a rapidly growing body of literature supports a broader role for N4BP1 in negatively regulating chemokine expression (19, 20). Notably, N4BP1 negatively regulates CXCL1 in the psoriatic skin by directly degrading its mRNA via its endoribonuclease activity, thereby limiting neutrophil infiltration (20). Given the highly conserved nature of CXC chemokines, it is plausible that N4BP1 similarly suppresses CXCL6, consistent with our single−cell expression data. These convergent functional and transcriptomic observations suggest that N4BP1 exerts a dual regulatory effect: it dampens both a pro−inflammatory axis (CXCL−CXCR) and, intriguingly, an anti−inflammatory axis (ANXA1−FPR). This functional duality suggests that N4BP1 in LDN-1 may fine−tune inflammatory responses rather than simply suppressing or promoting them.

## Discussion

Periodontitis is classified as a progressive disease that, if left untreated, can lead to tooth loss and exert adverse effects on overall health (21, 22). The progression of periodontitis is a complex process primarily driven by the aberrant actions of immune cells, particularly neutrophils. Neutrophil infiltration into periodontal tissues is a hallmark of periodontitis, reflecting an active immune response against periodontal pathogens. During inflammation or infection, inflamed tissues liberate key host-derived neutrophil chemoattractants, including tumor necrosis factor-α (TNF-α), interleukin-1β (IL-1β), IL-17, leukotrienes, prostaglandins, and complement component C5a (23). Concurrently, pathogen-derived mediators such as peptidoglycan and phenol-soluble modulins (24) further amplify neutrophil recruitment. These mediators drive the homing of mature neutrophils through postcapillary venules, a process termed neutrophil recruitment (25). Following infection resolution, neutrophils exit inflammatory sites under homeostatic conditions via a phenomenon termed “reverse migration”. This neutrophil reverse migration mechanism, previously characterized in research (26), enables neutrophils to migrate back into the vascular system from inflamed tissues. This process resolves infection while preventing collateral tissue damage caused by persistent neutrophil activation (27). Despite these insights, it remains unclear whether defined neutrophil subpopulations, including low−density neutrophils (LDNs), participate in specialized epithelial crosstalk in the context of periodontitis.

In this study, we integrated single−cell transcriptomics with Mendelian randomization to identify N4BP1 as a critical molecular regulator of neutrophil−epithelial crosstalk in periodontitis. Our central finding is the cell−type−specific and functionally dual role of N4BP1: its downregulation in gingival epithelial cells disrupts mucosal barrier integrity, while its upregulation in a transcriptionally defined LDN−like subset (LDN−1) modulates neutrophil−driven inflammatory signaling. This functional duality positions N4BP1 as a context−dependent rheostat that orchestrates immune−epithelial homeostasis, rather than a simple on−off inflammatory switch.N4BP1 functions as a cell−type−specific barrier protector in gingival epithelium. Our data show that N4BP1 is constitutively expressed in healthy gingival epithelial cells, and its downregulation during periodontitis correlates with reduced occluding (OCLN) (17, 28) and increased KRT5 (29, 30) expression—a signature of compromised epithelial integrity. Functional knockdown of N4BP1 in primary human gingival epithelial cells phenocopied these barrier defects, directly confirming a causal role. This epithelial−restricted function of N4BP1 aligns with its known role in controlling keratinocyte biology: in psoriatic skin, N4BP1 negatively regulates keratinocyte proliferation by degrading JunB and FosB mRNAs, thereby restricting epidermal hyperplasia (20). However, our study uncovers a previously unappreciated dimension—namely, that N4BP1 also governs barrier maintenance proteins such as occludin, thus bridging its functions in both proliferation control and barrier integrity. The loss of N4BP1 during periodontitis therefore likely renders the gingival mucosa more vulnerable to bacterial challenge and inflammatory insult.

In neutrophils, N4BP1 exerts a dual, net−anti−inflammatory effect on chemokine and pro−resolving pathways. Our scRNA−seq and CellPhoneDB analyses revealed that LDN−1 engages epithelial cells predominantly through the CXCL1/6−CXCR1/2 and ANXA1−FPR1/FPR2 axes. Functional experiments showed that pharmacological stabilization of N4BP1 in primary human neutrophils significantly reduced ANXA1 secretion, while single−cell data indicated lower CXCL1/6 expression in N4BP1^+^ cells. These observations, together with a rapidly growing body of literature, support a model in which N4BP1 acts as a negative regulator of both pro−inflammatory and pro−resolving signals. In psoriatic skin, N4BP1 directly degrades CXCL1 mRNA via its endoribonuclease activity, thereby limiting neutrophil infiltration (20). A recent study further established that N4BP1 suppresses the translation of Act1, a key adaptor in IL-17 signaling, leading to reduced expression of CXCL1, CCL20 and MMP9 (31). Moreover, N4BP1 has been shown to limit inflammatory cytokine responses in macrophages by constraining TRIF-independent TLR signaling (32). Collectively, these findings establish N4BP1 as a broadly conserved negative regulator of chemokine expression. However, our study reveals an intriguing additional layer: N4BP1 also suppresses ANXA1, a key anti-inflammatory mediator that signals through FPR1/FPR2 to resolve inflammation (33). In periodontitis, the ANXA1-FPR2 axis has been shown to play an unequivocally protective role, as genetic or pharmacological disruption of this pathway exacerbates alveolar bone resorption whereas administration of an ANXA1 mimetic peptide mitigates it (34). Thus, by suppressing both CXCL-mediated neutrophil recruitment and ANXA1-mediated resolution, N4BP1 in LDN-1 calibrates the balance between opposing inflammatory forces—ensuring that neutrophil activity does not escalate into uncontrolled tissue damage, albeit potentially at the expense of limiting endogenous pro-resolving signals.

The LDN−1 subset identified in this study contributes to a growing recognition of neutrophil heterogeneity as a driver of periodontal immunopathology. Low−density neutrophils have recently received attention for their roles in cancer, autoimmune diseases and inflammatory disorders (35). Notably, LDNs have now been experimentally linked to periodontitis: a 2024 case−control study demonstrated that LDN counts are significantly increased in periodontitis patients compared with healthy controls and are positively correlated with all periodontal parameters (36). In rheumatic diseases and systemic lupus erythematosus, LDNs are abnormally expanded and contribute to tissue damage through the release of neutrophil extracellular traps and pro−inflammatory cytokines (37, 38). Our study extends these observations by showing that a transcriptionally defined LDN−like subset (LDN−1) is specifically expanded in periodontitis and exhibits selective crosstalk with epithelial cells, thereby establishing a direct connection between LDN heterogeneity and tissue−specific immunopathology in the oral mucosa. This is consistent with a very recent single−cell atlas of human gingiva, which also identified a NETs−related neutrophil subpopulation and highlighted the importance of neutrophil−stromal crosstalk in periodontitis (39).

The present findings suggest that N4BP1 may serve as a promising therapeutic target. Its dual regulation—protecting epithelial barriers while limiting neutrophil−driven inflammation—argues for strategies that restore or enhance N4BP1 function in diseased tissues. However, the paradoxical suppression of the ANXA1−FPR pro−resolving pathway cautions that therapeutic modulation must be carefully calibrated: augmenting N4BP1 activity might dampen excessive neutrophil recruitment at the cost of impairing resolution signals. A more precise approach might therefore involve targeting the downstream pathways independently regulated by N4BP1, such as inhibiting CXCL1/6−CXCR1/2 while preserving ANXA1 activity. Such pathway−selective interventions could achieve the desired anti−inflammatory effect without compromising endogenous resolution programs.

Although periodontal therapy alleviates clinical inflammation, the early post-treatment phase is characterized by active tissue remodeling and immune cell repopulation. The increased epithelial–neutrophil interactions observed in the PDT group warrant careful contextualization. Initial periodontal therapy induces a rapid transition from active tissue destruction to wound healing and immune reconstitution. Within the first month, the gingival mucosa undergoes epithelial proliferation, stromal remodeling, and replenishment of the neutrophil pool. We propose that the enhanced interactions between epithelial cells and neutrophils in PDT (**Figure 1E**) represent repair−associated communication, primarily driven by Annexin A1−mediated pro−resolving signals and CSF3−dependent neutrophil homeostasis (**Figure 6A**), rather than the pathogenic chemokine−driven recruitment seen in untreated periodontitis. This interpretation supports the concept that successful periodontal therapy re−establishes immune–epithelial homeostasis through qualitatively distinct—albeit quantitatively robust—intercellular dialogues.

Furthermore, our findings reveal an apparent contradiction between global interactions and N4BP1 inhibition. Specifically, this contradiction is manifested as a marked discrepancy between the globally enhanced neutrophil−epithelial interactions in PD and the inhibitory effect of N4BP1 in LDN−1. To address this, we discuss two aspects: the population level and the cell−intrinsic regulatory level. At the population level, LDN-1 is markedly expanded in PD and represents the primary neutrophil subset engaging epithelial cells, thereby increasing the total communication pool. At the cell-intrinsic level, N4BP1 functions as a negative-feedback rheostat: within LDN-1, N4BP1^+^ cells exhibit attenuated per-cell communication probabilities compared with N4BP1^-^ counterparts, and N4BP1-high neutrophils show reduced CXCL1/6 expression. Thus, N4BP1 upregulation in LDN-1 likely represents a compensatory mechanism to restrain excessive inflammatory crosstalk, rather than a driver of it. In the absence of this N4BP1-mediated brake, pathogenic neutrophil–epithelial communication might be even more severe.

Several limitations of this study warrant consideration. First, the number of LDN−1 cells in the PD group (n = 17) was modest, limiting the statistical power to detect subtle expression differences, including the observed trend of N4BP1 upregulation. Bootstrap analysis yielded a 95% confidence interval for the expression difference that crossed zero (-0.295 to 1.263), indicating that the single−cell evidence for increased N4BP1 in LDN−1 should be interpreted cautiously. Nevertheless, the subsequent random down-sampling analysis still supports the main conclusions of the study (**Supplementary Figures 1, S6 and S7**). Second, the LDN−1 subset was annotated solely by a reference−based computational method (SingleR); orthogonal validation by flow cytometry or CITE−seq is therefore required to confirm the identity and surface phenotype of this subset. Third, the murine ligature−induced periodontitis model, despite its wide use and reproducibility, does not fully recapitulate the chronic, polymicrobial and patient−heterogeneous nature of human periodontitis. Future studies using longitudinal human cohorts and conditional N4BP1 knockout mice will be essential to establish the precise temporal and cell−type−specific contributions of N4BP1 in periodontitis progression.

In conclusion, this work establishes N4BP1 as a cell−type−specific molecular switch that coordinates epithelial barrier function and neutrophil−mediated inflammation in periodontitis. The identification of the LDN−1 subset expands our appreciation of neutrophil heterogeneity in oral pathology and positions N4BP1 as a candidate for future mechanism−based therapeutic strategies.

## Materials and methods

### Single-cell transcriptome analysis

#### Study design and data sources

This study employed a combination of single-cell RNA sequencing (scRNA-seq) and bidirectional Mendelian randomization (MR) analyses to investigate the role of neutrophils and the N4BP1 molecule in the progression of periodontitis. The scRNA-seq data were obtained from periodontal tissues of healthy controls (HC, n=4, patients with severe chronic periodontitis (PD, n=5), and patients with severe chronic periodontitis after initial periodontal therapy within 1 month (PDT, n=3) (40). The single-cell data were processed and analyzed to identify cell populations and their interactions, with a particular focus on neutrophils and epithelial cells. For the MR analysis, we utilized summary-level data from the IEU OpenGWAS database (41), specifically the datasets ebi-a-GCST90018897, ebi-a-GCST90018677, and finn-b-K11_GINGIVITIS_PERIODONTAL, to assess the causal relationships between differentially expressed genes and periodontitis.

#### Data preprocessing and quality control

Quality control (QC) was performed to filter out low-quality cells and genes. Cells with fewer than 200 detected genes or more than 5% mitochondrial gene content were excluded. Genes expressed in fewer than 3 cells were also removed. After QC, a total of 2507 single cells were retained, including 943 cells from HCs, 442 cells from PDs, and 1122 cells from PDTs.

#### Cell clustering and annotation

The filtered gene expression matrices were normalized using the Seurat package (version 4.4.0) (42). Principal component analysis (PCA) was performed to reduce dimensionality, and the top 20 principal components were used for clustering. Uniform Manifold Approximation and Projection (UMAP) was employed for visualization. Cell types were annotated using the online platform CellMarker 2.0 (43) based on canonical marker genes: Plasma cells (characterized by MZB1, DERL3, XBP1), T cells (TRAC, CD2, IL32, CCL5), Monocytes (HLA-DPB1, HLA-DRA, CD74), Neutrophils (FCGR3B, CSF3R, CXCR1, G0S2), Epithelial cells (KRT5, KRT14, TACSTD2), Stromal cells (COL1A2, COL3A1, SPARC, FN1), Keratinocytes (SPRR1A, SPRR3, KRT6C, KRT4), and Endothelial cells (PECAM1, CDH5, KDR, CD34). The expression patterns of these marker genes across clusters confirmed the accuracy of cell type identification. Using the same approach, we performed subclustering and annotation of the neutrophil populations and identified five neutrophil subsets: Interferon-stimulated neutrophils, a transcriptionally defined LDN−like subset (designated LDN−1), Classical neutrophils, another LDN−like subset (LDN−3), and Inflammatory neutrophils. Among these, LDN-1 and LDN-3 were both annotated by SingleR (44) as transcriptionally resembling low−density neutrophils, although density−based isolation was not performed.

### Differential gene expression analysis

Using the FindMarkers function in Seurat, we identified genes that are differentially expressed between LDN-1 and other cell populations, including other neutrophil subsets. We retained all differentially expressed genes associated with LDN-1 and subsequently employed Mendelian randomization (MR) and single-cell analysis to identify and screen for key genes involved in the progression of periodontitis.

### Cell-cell interaction analysis

CellPhoneDB (version 1.6.1) (45) was used to infer cell-cell interactions based on ligand-receptor pairs. The analysis was performed separately for the HC, PD, and PDT groups to identify changes in intercellular communication during disease progression and treatment. The results were visualized using circle plots to highlight significant interactions between epithelial cells and neutrophils.

### Mendelian randomization analysis

#### Data sources and instrument selection

For the MR analysis, we utilized summary-level data from the IEU OpenGWAS database (41). The datasets included ebi-a-GCST90018897, ebi-a-GCST90018677, and finn-b-K11_GINGIVITIS_PERIODONTAL. Genetic variants (single nucleotide polymorphisms, SNPs) associated with the expression of candidate genes were selected as instrumental variables (IVs). SNPs were required to have a genome-wide significance threshold of p < 5 × 10^-8 and a linkage disequilibrium (LD) threshold of r² < 0.001 within a 10,000 kb window. JAK3 was excluded from primary consideration because it was supported by only a single instrumental variable, precluding robust sensitivity analyses.

#### MR analysis methods

The MR analysis was conducted using the TwoSampleMR R package (version 0.6.3) (44). We employed the inverse-variance weighted (IVW) method as the primary approach to estimate causal effects. Sensitivity analyses were performed using the MR-Egger method (to assess directional pleiotropy) and the weighted median method (which provides valid estimates if ≥50% of the weight comes from valid IVs). Heterogeneity was evaluated using Cochran’s Q test, and horizontal pleiotropy was assessed using the MR-Egger intercept test (**Table 2** and **Table 3**).

#### Gene expression validation

The expression of candidate genes identified through MR analysis was validated using the scRNA-seq data. The expression levels of N4BP1, UBE2D1, MYL6, MAPK14, MCEMP1, and LILRA5 were compared across different cell types and conditions (HC, PD, PDT). The results were visualized using dot plots and violin plots to illustrate the differential expression patterns.

### Functional enrichment analysis

Gene ontology (GO) and Kyoto Encyclopedia of Genes and Genomes (KEGG) pathway analyses were performed using the OmicShare tools (46). Differentially expressed genes identified in the LDN-1 were analyzed to identify enriched biological processes and pathways. The results were visualized using bubble plots and enrichment circle diagrams to highlight the most significantly enriched terms.

### Animal experiments

#### Mice

All animal experiments were performed in accordance with the ARRIVE guidelines 2.0. Animal experimental procedures were approved by the Laboratory Animal Care and Use Committee at Anhui Medical University (Approval No. LLSC20220738). The eight-week-old male C57BL/6 mice were utilized for all experiments. Eight-week-old male C57BL/6 mice were housed under specific pathogen-free (SPF) conditions (temperature 22 ± 1°C, humidity 50 ± 10%, 12 h light/dark cycle) with ad libitum access to standard rodent chow and water. Mice were randomly assigned to two groups (n = 7 per group): (1) Control group; (2) Periodontitis group (Suture ligation of the maxillary right second molar in mice).

#### Murine periodontitis model

The silk ligature-induced periodontitis model was selected for its high reproducibility, rapid induction of alveolar bone loss within two weeks, and established utility in studying immune and stromal interactions. Periodontitis was induced in 8-week-old male C57BL/6 mice (n = 7/group) via ligature placement around the maxillary right second molars. In detail, under induction of anesthesia with intraperitoneal injection of 2% pentobarbital (50 mg/kg), a sterilized (autoclaved) 5–0 silk suture was passed subgingivally around the neck of the right maxillary second molar, engaging both the mesial and distal interdental papillae, and securely tied with a surgeon’s knot. Ligatures were visually inspected every 2–3 days; if loosened or lost, they were immediately replaced under the same anesthetic regimen. Ligatures remained in place for two weeks to induce periodontal bone loss.

#### Sample collection and processing

On postoperative day 15, all mice were deeply anesthetized with an intraperitoneal injection of 2% pentobarbital (50 mg/kg), and blood samples were collected via intracardiac puncture. The mice were then immediately euthanized by cervical dislocation, and gingival tissues as well as maxillae were harvested for subsequent analyses. Fresh blood was collected and stored in EDTAK2 anticoagulant tubes (Kangweishi, China) within 2h. Neutrophils were isolated from blood using a commercially available Mouse Peripheral Blood Neutrophil Isolation Kit (P9201, Solarbio) following the manufacturer’s protocol. Gingival tissues were homogenized in RIPA Lysis Buffer (Beyotime) for protein extraction. Maxillae were dissected and fixed in 4% paraformaldehyde for 24–48 hours. The fixed maxillae were then subjected to micro-CT analysis. After micro-CT scanning, the maxillae were decalcified in 10% EDTA (pH 7.4) for histological processing (decalcification, paraffin embedding, sectioning, and staining).

### Protein extraction and western blots

The lysates were incubated on ice for 20 min with agitation, followed by centrifugation at 13,000 rpm and 4°C for 10 min. T The supernatant was transferred to a new 1.5 mL microcentrifuge tube, mixed 1:1 with 2× SDS-PAGE loading buffer (Beyotime) containing protease and phosphatase inhibitors, boiled at 95°C for 10 min, and stored at -20°C or directly subjected to Western blot analysis. Protein samples were separated on 10% Bis-Tris gel, and then wet transferred to a 0.45 µm pore size membrane (Merck) in ice-cold transfer buffer for 2h. After blocked with 5% non-fat milk for 1h at room temperature, the membrane was incubated with the primary antibody overnight at 4°C. After washed four times with TBST for 7 min, the membrane was incubated with HRP-conjugated secondary antibodies for 1h at room temperature. After washed four times with TBST each wash for 5 min. The membrane was imaged using a CCD camera for visualizing the immunoreactive bands using enhanced chemiluminescence detection (Tanon, Shanghai, China). Primary antibodies included Rabbit polyclonal antibody to N4BP1 (DF4222, Affinity, 1:1000), OCLN (27260-1-AP, Proteintech, 1:1000) and KRT5 (66727-1-IG, Proteintech, 1:2000). GAPDH (60004-1-IG, Proteintech, 1:2000) served as the loading control. Band intensities were quantified using ImageJ, with grayscale values normalized to GAPDH.

### Epithelial cell line culture and N4BP1 knockdown

Primary human gingival epithelial cells (PGECs) were isolated from healthy gingival tissues obtained during routine third molar extractions (approved by the Institutional Review Board, with informed consent). Cells were cultured in keratinocyte−serum free medium (KSFM, Gibco) supplemented with 50 μg/mL bovine pituitary extract (BPE) and 5 ng/mL epidermal growth factor (EGF) at 37 °C in 5% CO_2_. For N4BP1 knockdown, cells were transfected with small interfering RNA (siRNA) targeting human N4BP1 (GenePharma, Shanghai, China) using Lipofectamine 3000 (Invitrogen) according to the manufacturer’s instructions. All siRNAs were synthesized by GenePharma with HPLC purification and used at a final concentration of 50 nM. After 48 h of transfection, cells were harvested and lysed in RIPA buffer (Beyotime) supplemented with protease and phosphatase inhibitors. Protein lysates were subjected to western blot as described above. Primary antibodies against N4BP1 (DF4222, Affinity, 1:1000), OCLN (27260-1-AP, Proteintech, 1:1000), KRT5 (66727-1-IG, Proteintech, 1:2000), and β-actin (20536-1-AP, Proteintech, 1:1000) were used. The experiment was repeated three times independently.

### Neutrophil isolation, MG-132 treatment and ANXA1 ELISA

Human peripheral blood (20 mL) was collected from healthy volunteers into EDTA−containing tubes (Kangweishi, China). Neutrophils were isolated immediately using a Human Peripheral Blood Neutrophil Isolation Kit (P9201, Solarbio) following the manufacturer’s protocol. Isolated neutrophils were resuspended in RPMI 1640 medium (Gibco) supplemented with 10% fetal bovine serum (FBS) and 1% penicillin-streptomycin. To elevate endogenous N4BP1 protein levels, cells were treated with the proteasome inhibitor MG-132 (CAS 133407-82-6, Santa Cruz Biotechnology, sc-201270) at a final concentration of 20 μM for 3h at 37 °C. MG-132 is a cell-permeable proteasome inhibitor that prevents ubiquitin-dependent protein degradation. This pharmacological approach was selected because N4BP1 contains a ubiquitin-associated (UBA) domain and undergoes rapid proteasomal turnover; MG-132 stabilization thus preserves native post-translational modifications and regulatory interactions, offering advantages over genetic overexpression strategies.

An equivalent volume of DMSO was added to control cells. After treatment, the cell culture supernatant was collected and centrifuged at 1,500 × g for 10 min to remove debris. The cleared supernatant was stored at –80 °C for subsequent ELISA. The cell pellets were washed twice with ice-cold PBS and lysed in RIPA buffer (Beyotime) for western blot analysis of N4BP1 (DF4222, Affinity, 1:1000). The concentration of human Annexin A1 (ANXA1) in the supernatant was measured using a commercial Human ANXA1 ELISA Kit (CSB-E12155h, CUSABIO, China) according to the manufacturer’s protocol. Each sample was assayed in duplicate, and the experiment was performed with three independent biological replicates.

### Microcomputed tomography analysis and histological observation

Maxillary specimens were fixed in 4% paraformaldehyde (24h, 4°C) and stored in 75% ethanol. Scans were acquired using a high-resolution SkyScan 1176 system (Bruker, Kontich, Belgium; 50 kV, 0.5 mm Al filter, 18 μm isotropic resolution). The region of interest (ROI) was defined as the alveolar bone surrounding the second maxillary molar. 3D reconstructions were generated in CTvox (v3.3.0.0) using standardized thresholding (grayscale 80-255). Cementoenamel junction-to-alveolar bone crest (CEJ-ABC) distance and buccal bone loss area were quantified in ImageJ (v1.54g) via semi-automated segmentation.

Post-micro-CT, specimens were decalcified in 10% EDTA (pH 7.4, 4°C) for 4 weeks (decalcification confirmed by radiography). Tissues were paraffin-embedded, sectioned coronally at 4 μm thickness, and stained with hematoxylin and eosin (H&E) to evaluate bone morphology.

### H&E staining

Tissue sections (4 μm) were subjected to H&E staining using standardized protocols. Briefly, deparaffinized sections were stained with Mayer’s hematoxylin (4 min), differentiated (1% acid ethanol, 3 sec), blued (0.2% ammonia water), and counterstained with eosin Y (3 min). After dehydration and mounting, slides were imaged using a Nikon Eclipse E100 microscope.

### Immunofluorescence staining for paraffin-embedded tissues

Tissue sections were deparaffinized in xylene (2 × 15 min) and rehydrated through graded ethanol series (100%, 100%, 85%, 75%; 5 min each) followed by distilled water rinse. Antigen retrieval was performed in citrate buffer (10 mM, pH 6.0) using microwave heating (95°C, 20 min). After natural cooling, slides were washed in PBS (3 × 5 min).

Tissue sections were encircled with a hydrophobic barrier, treated with autofluorescence quencher (Servicebio) for 5 min, and rinsed under running water (10 min). Non-specific binding was blocked with 3% BSA/PBS (30 min, RT). Primary antibodies (diluted in PBS) were applied and incubated overnight at 4°C in a humidified chamber. After PBS washes (3 × 5 min), fluorophore-conjugated secondary antibodies (species-matched; Servicebio) were applied (50 min, RT, dark). Nuclei were counterstained with DAPI (1 µg/mL, 10 min, dark). Sections were mounted with anti-fade medium (Servicebio, G1401) and imaged using a fluorescence microscope (Nikon Eclipse Ni-E) with standard filter sets:DAPI: Ex 330–380 nm/Em 420 nmFITC: Ex 465–495 nm/Em 515–555 nmCy3: Ex 510–560 nm/Em 590 nm.

### Statistical analysis

All non-experimental statistical analyses were conducted using R software (version 4.3.2). For scRNA-seq data, differential expression analysis was conducted using the Wilcoxon rank-sum test. For MR analysis, p-values < 0.05 were considered statistically significant. Sensitivity analyses were performed to ensure the robustness of the MR results. All experimental data were analyzed using GraphPad Prism (v9, GraphPad Software), with results expressed as mean ± standard error of the mean (SEM) derived from at least three independent biological replicates. For two-group comparisons of normally distributed data (confirmed by Shapiro-Wilk test), unpaired two-tailed Student’s t-tests were used. Non-parametric data were analyzed using Mann-Whitney U tests. Data between multiple groups were compared with one-way analysis of variance (ANOVA) followed by the Tukey’s *post hoc* test. The specific P values are detailed in the figure legends and were considered statistically significant at P < 0.05.

## Data Availability

The original contributions presented in the study are included in the article/**Supplementary Material**. Further inquiries can be directed to the corresponding authors.
