# Supplementary material for: Dual role of N4BP1 in neutrophil–epithelial crosstalk in periodontitis
Source: Front Immunol. 2026 May 28;17:1830039. doi: 10.3389/fimmu.2026.1830039 (PMC13253295; doi:10.3389/fimmu.2026.1830039)
Supplement: Supplementary file 1 [file DataSheet1.docx]

**Supplementary figures legend**

**Figure S1. Downsampling sensitivity analysis of cell-type proportions.** (a–b) Violin plots showing epithelial cell proportions after downsampling HC and PDT to n = 442 cells (100 repetitions and 1,000 repetitions). (c–d) Violin plots showing neutrophil proportions after downsampling HC and PDT to n = 442 cells (100 repetitions and 1,000 repetitions).Red crosses indicate original proportions.

**Figure S2. Detection probability of the LDN-1 subset.** Line plot showing the probability of detecting the LDN-1 subset in the PD group as a function of total neutrophil count. The probability reaches 1.0 when the total neutrophil count exceeds approximately 10 cells.

**Figure S3. Sensitivity analyses for Mendelian randomization of periodontitis-associated genes.**

(a) Leave-one-out sensitivity analyses for candidate genes. Each plot shows the IVW estimate when sequentially excluding individual SNPs. (b) Leave-one-out analyses. Horizontal lines in leave-one-out plots represent the full IVW estimate. IVW, inverse variance weighted.

**Figure S4. Expression validation of periodontitis-associated genes across cell types and conditions.**

(a-e) Violin plots show transcript levels of MR-identified candidate genes (UBE2D1, MAPK14, MCEMP1, LILRA5, MYL6) in gingival tissues. MR, mendelian randomization.

**Figure S5. KEGG enrichment analysis of N4BP1-associated functions.**

**Figure S6. Robustness of cell-cell communication probabilities.** (A) Boxplots of ANNEXIN and CXCL pathway communication probabilities (CellChat) across 10 subsampling repeats. (B) Boxplots of specific ligand–receptor pair probabilities (CXCL1/6–CXCR1/2, ANXA1–FPR1/2) across 10 repeats.

**Figure S7. Stability of N4BP1 expression across subsampling repeats.** (a) Boxplots of N4BP1 expression in epithelial cells and neutrophils across 50 subsampling repeats (HC, PD, PDT). (b) Boxplots of N4BP1 expression in epithelial cells and neutrophils across 100 subsampling repeats (HC, PD, PDT).
