# Supplementary figures and images for: Dual role of N4BP1 in neutrophil–epithelial crosstalk in periodontitis
Source: Front Immunol. 2026 May 28;17:1830039. doi: 10.3389/fimmu.2026.1830039 (PMC13253295; doi:10.3389/fimmu.2026.1830039)

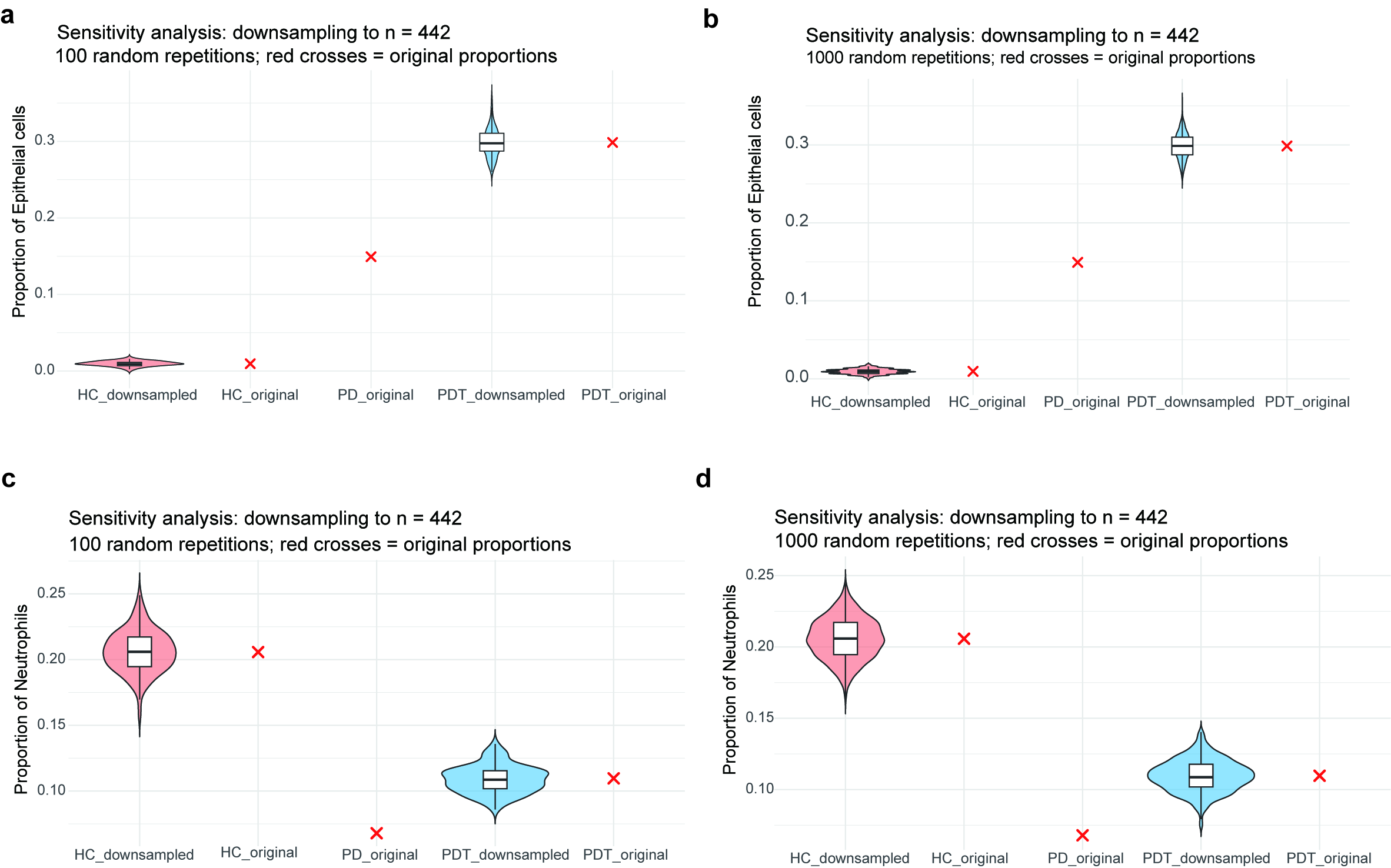

Supplement: Supplementary file 2 [file Image1.tif]

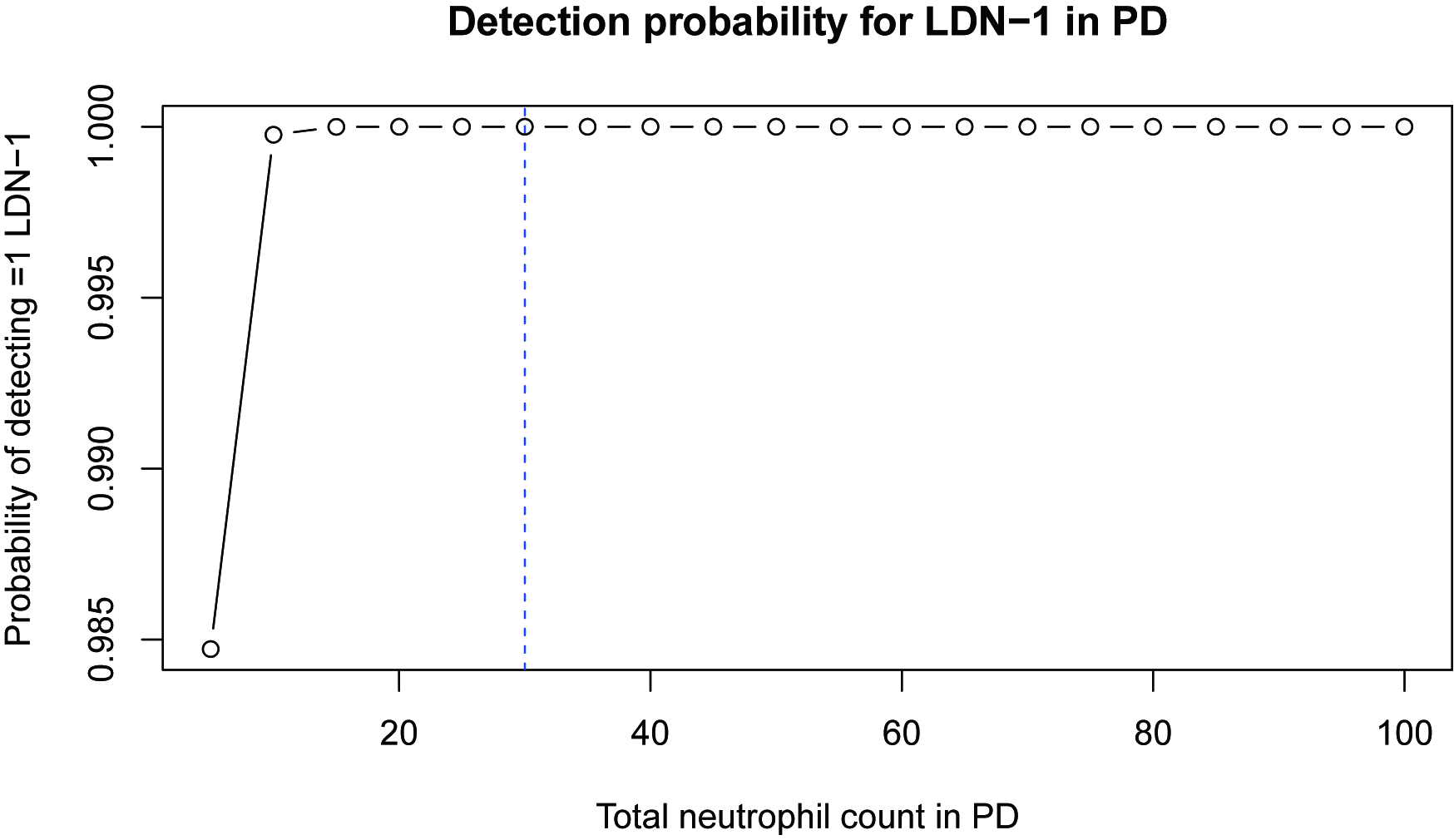

Supplement: Supplementary file 3 [file Image2.tif]

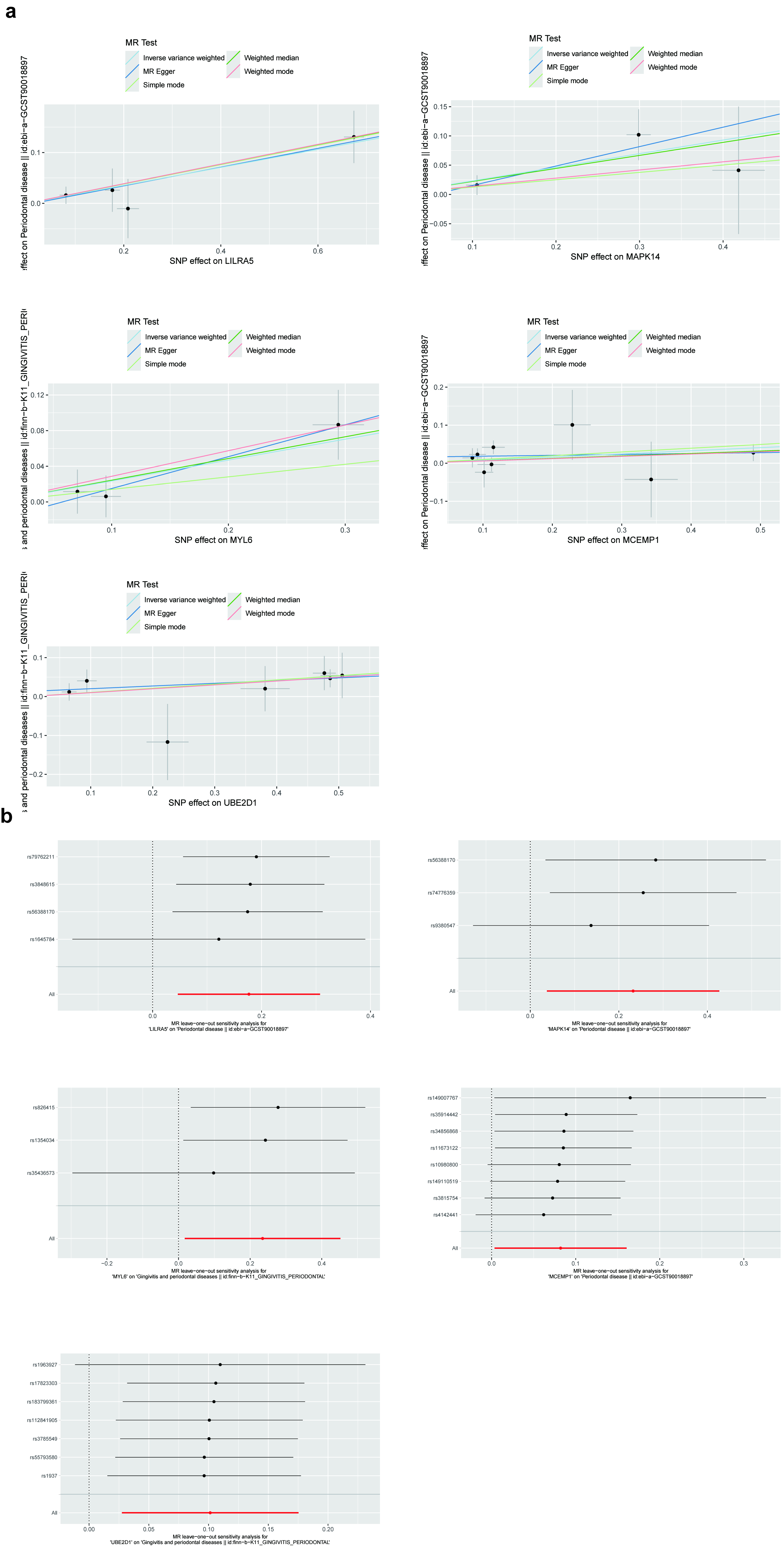

Supplement: Supplementary file 4 [file Image3.tif]

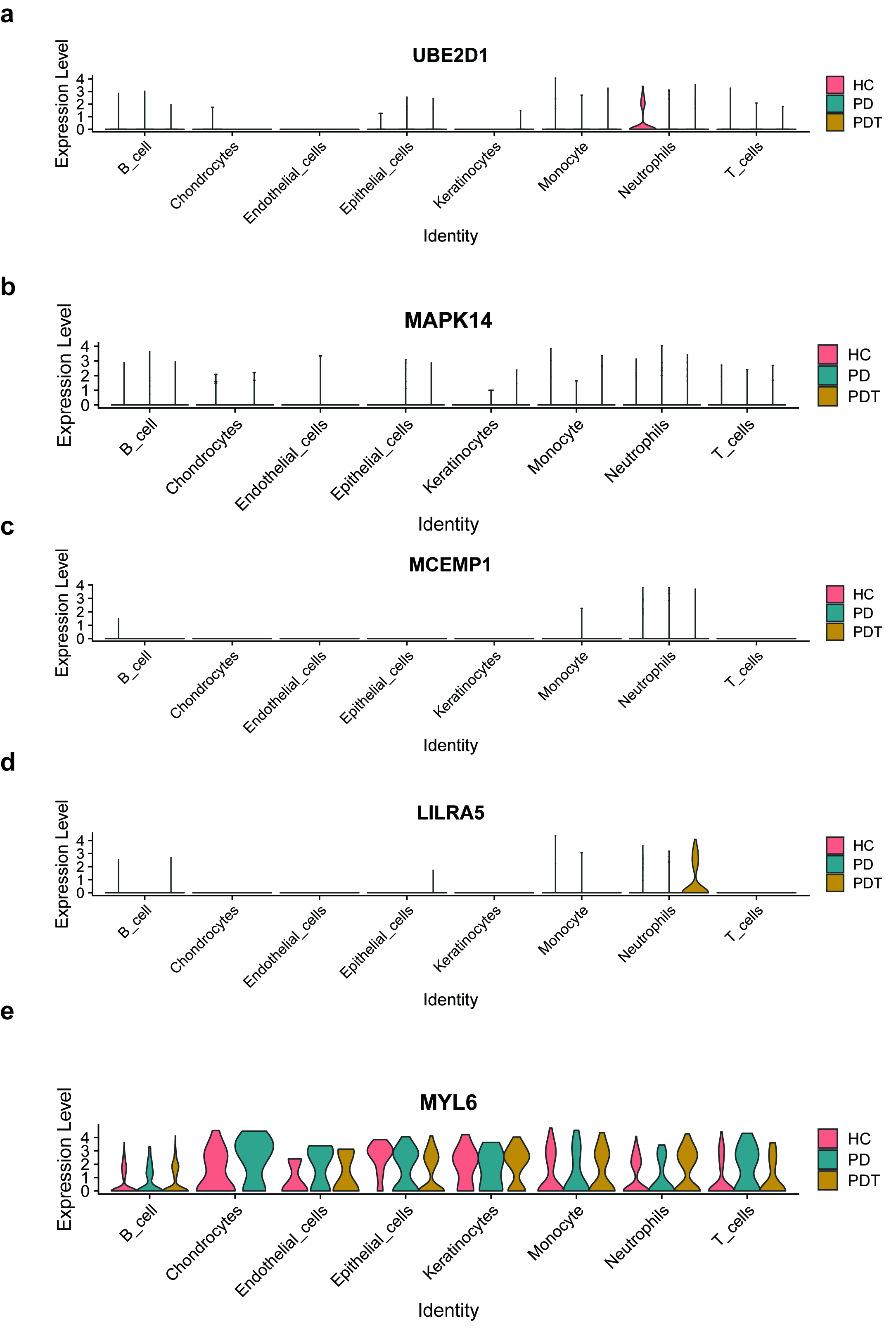

Supplement: Supplementary file 5 [file Image4.tif]

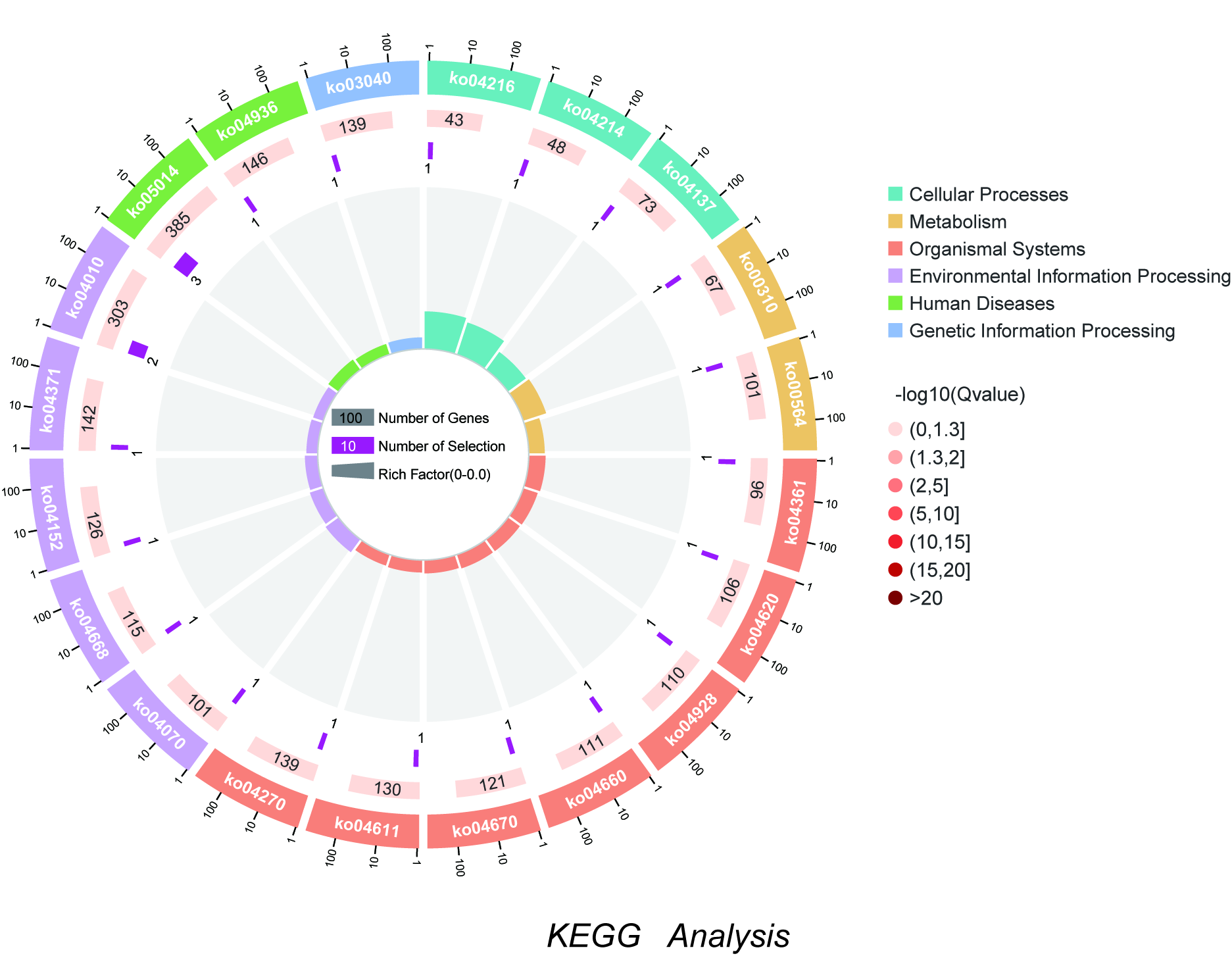

Supplement: Supplementary file 6 [file Image5.tif]

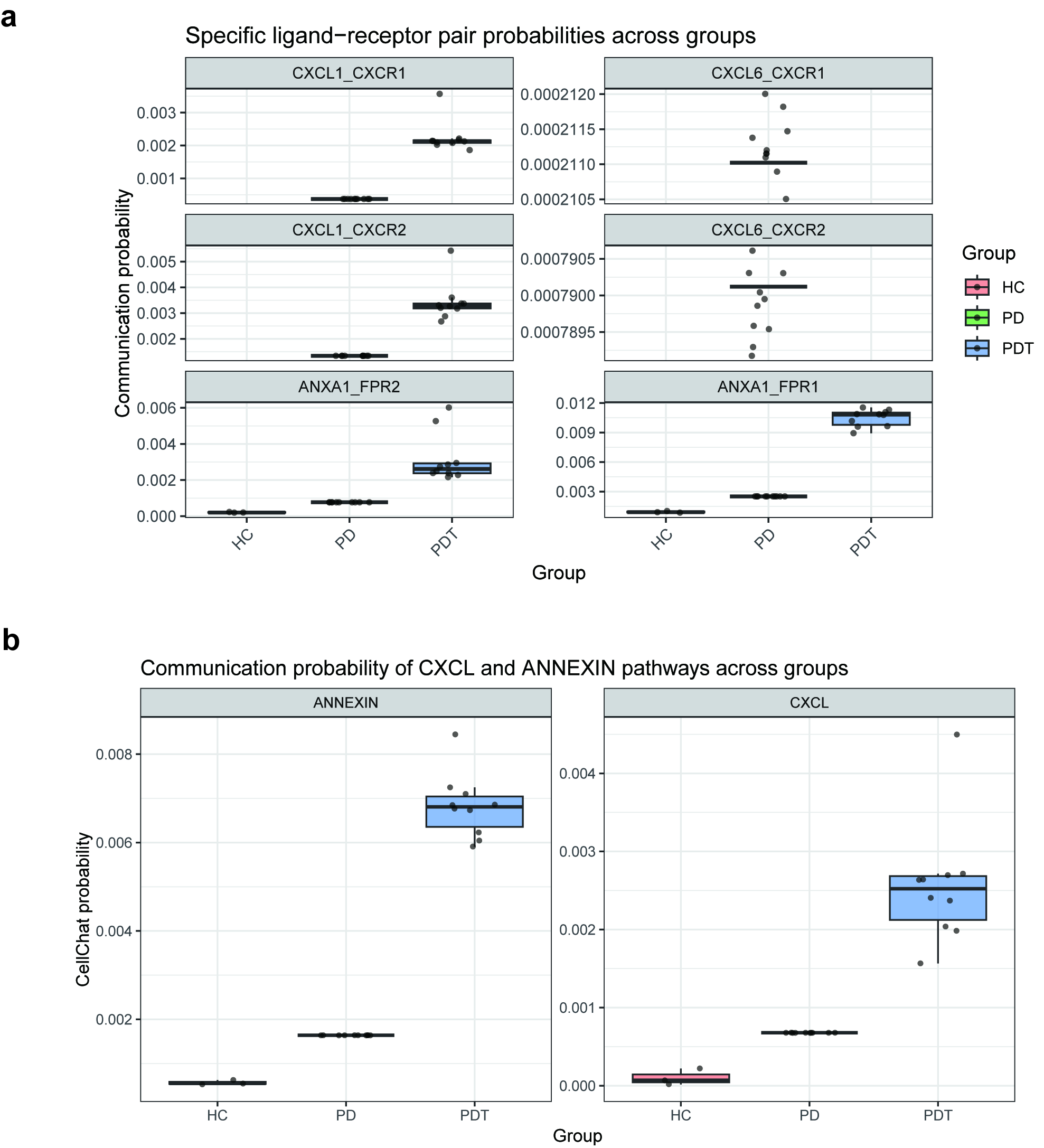

Supplement: Supplementary file 7 [file Image6.tif]

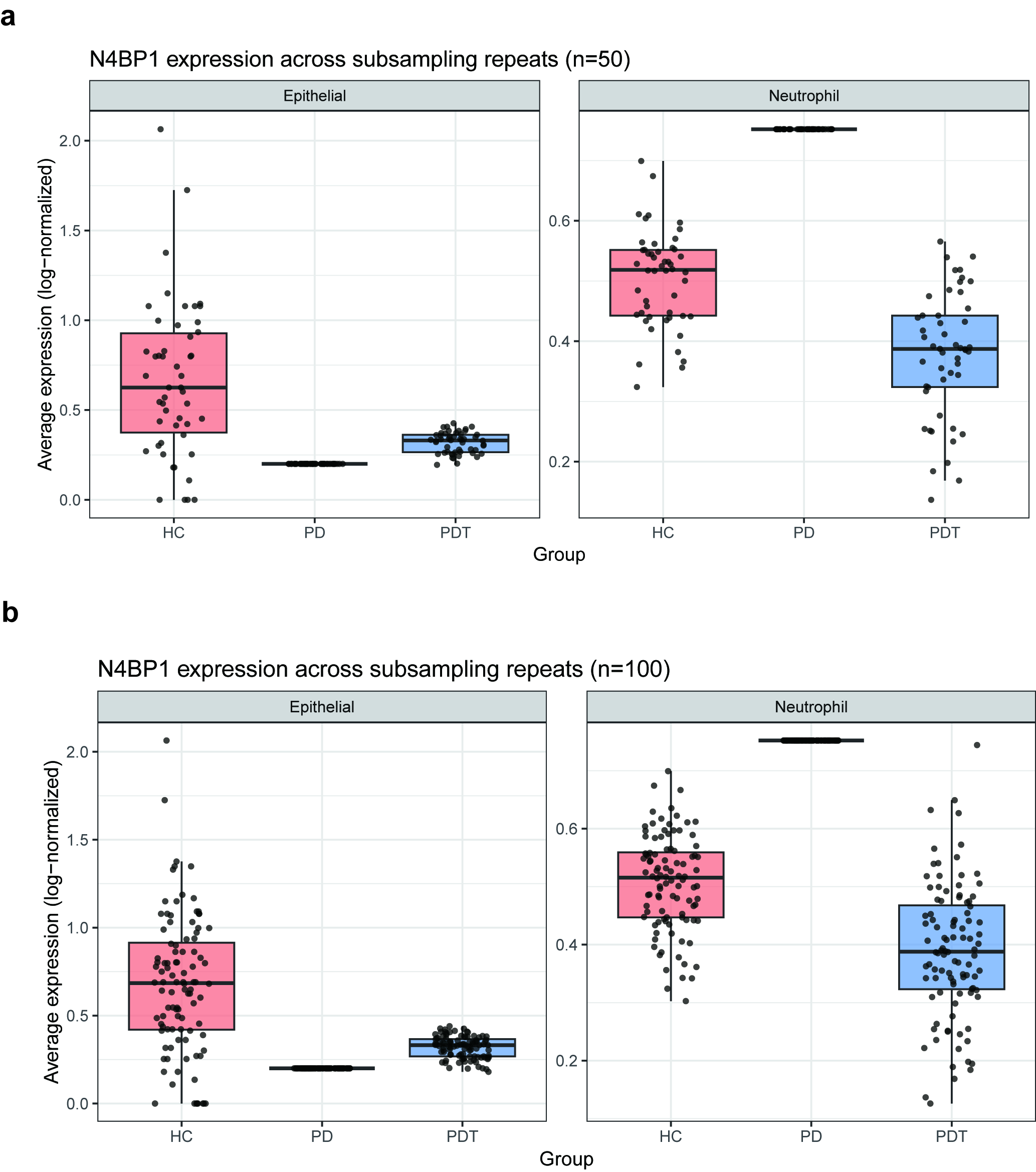

Supplement: Supplementary file 8 [file Image7.tif]
